# Advanced thermal sensing techniques for characterizing the physical properties of skin

**DOI:** 10.1063/5.0095157

**Published:** 2022-12

**Authors:** Surabhi R. Madhvapathy, Hany M. Arafa, Manish Patel, Joshua Winograd, Jessy Kong, Jason Zhu, Shuai Xu, John A. Rogers

**Affiliations:** 1 Department of Materials Science and Engineering, Northwestern University, Evanston, Illinois 60208, USA; 2 Querrey Simpson Institute for Bioelectronics, Northwestern University, Chicago, Illinois 60611, USA; 3 Department of Biomedical Engineering, Northwestern University, Evanston, Illinois 60208, USA; 4 University of Illinois College of Medicine at Chicago, Chicago, Illinois 60612, USA; 5 Department of Chemical Engineering, Northwestern University, Evanston, Illinois 60208, USA; 6 Department of Dermatology, Feinberg School of Medicine, Northwestern University, Chicago, Illinois 60611, USA

## Abstract

Measurements of the thermal properties of the skin can serve as the basis for a noninvasive, quantitative characterization of dermatological health and physiological status. Applications range from the detection of subtle spatiotemporal changes in skin temperature associated with thermoregulatory processes, to the evaluation of depth-dependent compositional properties and hydration levels, to the assessment of various features of microvascular/macrovascular blood flow. Examples of recent advances for performing such measurements include thin, skin-interfaced systems that enable continuous, real-time monitoring of the intrinsic thermal properties of the skin beyond its superficial layers, with a path to reliable, inexpensive instruments that offer potential for widespread use as diagnostic tools in clinical settings or in the home. This paper reviews the foundational aspects of the latest thermal sensing techniques with applicability to the skin, summarizes the various devices that exploit these concepts, and provides an overview of specific areas of application in the context of skin health. A concluding section presents an outlook on the challenges and prospects for research in this field.

## I. INTRODUCTION AND FRAMEWORK OF THE REVIEW

Interest in measuring the intrinsic properties of the skin arises from the need for quantitative assessments of dermatological health and for increasing our understanding of physiological processes that occur in underlying tissues. Specifically, the physical characteristics of skin reflect basic mechanisms that occur in this organ of the human body, such as those related to blood flow, thermoregulation, homeostasis, and barrier protection. Changes in these properties can reveal immunological disorders associated with skin graft rejections, anomalous hemodynamic responses relevant to peripheral artery disease (PAD), altered healing associated with burns and ulcers, and restricted flow due to venous thrombosis/chronic venous insufficiency and arterial aneurysms. Pathologies of the skin, ranging from malignancies (e.g., melanomas), autoimmune disorders (e.g., atopic dermatitis, or AD), to infectious conditions, such as herpes simplex and cellulitis, also lead to unique types of changes. In other contexts, measurements on the surface of the skin can yield insight into the behavior of superficial implants, such as shunts/catheters for hydrocephalus patients and arteriovenous fistulas for kidney dialysis, and into underlying body processes related to cardiopulmonary activity. Interest in precision measurements primarily follow from these and other health related topics, but relevance extends to basic scientific studies of the skin and various aspects of skin care and cosmetics.

The content of this review begins with a background and motivation for studying the thermal properties of the skin, including an overview of (Sec. II) the fundamental structure of the skin and its relation to disease states, techniques for characterizing the properties of the skin, and the thermal properties of the skin. Section III highlights measurements of skin surface temperature. Section IV reviews isotropic thermal transport properties of the skin, with specific emphasis on the transient plane source and 3*ω* sensing techniques. Section V examines dynamic fluid flow in the skin, with emphasis on thermal anemometry, calorimetry, and time-of-flight techniques. In each of these sections, an overview of the measurement physics precedes examples that showcase applications of these techniques with clinical relevance, including the assessment and monitoring of near-surface dermatological diseases (e.g., melanoma), skin hydration, hemodynamics, wound healing, and neurological disorders like hydrocephalus. The concluding section (Sec. VI) summarizes technology trends and presents some thoughts on future opportunities for the role of engineering in the health science of the skin.

## II. BACKGROUND AND MOTIVATION FOR STUDYING THE THERMAL PROPERTIES OF THE SKIN

### A. The structure of the skin

As the largest organ of the body, the skin comprises an average surface area of ∼ 2 m^2^ and accounts for ∼12%–16% of the total weight of an adult.^1–4^ The skin has multiple key functions: (1) thermoregulation, through the modulation of blood flow to the near surface regions of the skin and through the release of sweat; (2) vitamin D production, through the interaction of cholesterol precursors in the skin with UV radiation; (3) sensation to heat/cold, touch, pressure, vibration, and pain, through distributed collections of mechano- and thermoreceptors; (4) absorption of gases and medications through transcellular diffusion; (5) excretion of waste through the release of sweat; and (6) immunological functions providing resistance to microorganisms and noxious external agents.^3,5,6^ The three main layers of the skin—the epidermis, dermis, and hypodermis—act together in supporting these essential roles.

An overview of the essential structural features of the skin are described in Table I. The epidermis is thinnest on the eyelids and thickest on the soles of the feet.^7^ This outermost layer of the skin is avascular and is comprised primarily (∼95%) of cells that produce the keratin protein, known as keratinocytes.^5^ Corneocytes and an associated lipid matrix define the outermost layer of the epidermis, known as the stratum corneum. Corneocytes naturally retain water while lipids impede the escape/evaporation of water from the surface of the skin. Together, these components hinder water loss from the underlying tissues to the surroundings. Epidermal water resides both within the cells and within this extracellular lipid matrix. Loss of water can occur from this and underlying layers of the skin.

**TABLE I.** Key features of the skin influencing its thermal properties.

Layer	Thickness	Tissue/cellular components	Blood vessels	Appendages
Epidermis	Typically, ∼ 100 *μ*m, [range: ∼50 *μ*m (eyelids) −600 *μ*m (soles of feet)]^7^	Keratinocytes (95%),^5^ melanocytes, Merkel cells, Langerhans cells	⋯	Hair shafts, sweat pores, nails
Dermis	1–4 mm^7^	Collagen, elastin, fibrillin, fibroblasts, macrophages, mast cells	Capillaries (10–12 *μ*m diameter)^10^ venules/arterioles (12–25 *μ*m diameter)^10^ near-surface vein/arteries (∼2–4 mm diameter)^151^	Hair follicles 10–100 s cm^−2^ (Refs. 5, 17, and^18^) Sweat glands ≤∼600–700 cm^−2^ (palms and feet)^4^ Sebaceous glands ≤∼400–900 cm^−2^ (scalp and forehead)^16^
Hypodermis	2–7 mm^8^	Adipose tissue (adipocytes), collagen, elastin	Capillaries, venules/arterioles (50 *μ*m diameter),^10^ Arteries/veins	Sweat glands

The dermis is a highly vascularized layer that lies beneath the epidermis, comprised of connective tissue formed from collagen. The dermis is typically 1–4 mm in thickness across most body locations.^7^ Beneath the dermis lies the hypodermis, a 2- to 7-mm-thick layer comprising of fatty connective tissue that cushions the internal organs from external impacts and provides some thermal insulation against the surroundings.^8^ Blood flow through vessels in the dermis accounts for ∼5%–10% of the total cardiac output volume.^9^ This flow can occur as capillary microcirculation (microvascular flow or perfusion) or as macrovascular flow through veins and arteries. Capillaries are small blood vessels that support diffusive exchange with surrounding cells.^10^ Branched capillaries connect to veins and arteries through small corresponding vessels, called “venules” and “arterioles” that lead to superficial veins and arteries. Microcirculatory perfusion is the rate of isotropic, volumetric blood flow through a given mass or volume of tissue.^11^ In the dermis, perfusion (denoted by *w*) is typically 1.25 × 10^−3^ m^3^ s^−1^ m^−3^ tissue.^11^

The deeper layers of the epidermis and the entire dermis include both lymphatic and circulatory channels, aiding in blood delivery and immune response. Both lie within the extracellular matrix (ECM), a network of supportive connective tissue, and are surrounded by interstitial fluid (ISF). ISF constitutes 60%–70% of the overall fluid content of the body.^12,13^ In bulk tissue, volumetric ISF flow is largely isotropic; typical anisotropic lymphatic flow rates in vessels (>20 *μ*l/h) are negligible compared to those of blood. Depending on the diameter of the vessel, blood flow rates range from 0.1 ml/min (i.e., retinal vessels) to 600 ml/min (i.e., coronary arteries and jugular veins). These rates can, however, increase dramatically, for example, up to 6–8 l/min during episodes of hyperthermia.^14,15^ The dermis contains other important physiological features (appendages) including hair follicles, sweat glands, sebaceous glands, and cutaneous nerves.^4,5,16–18^

**FIG. 1.**
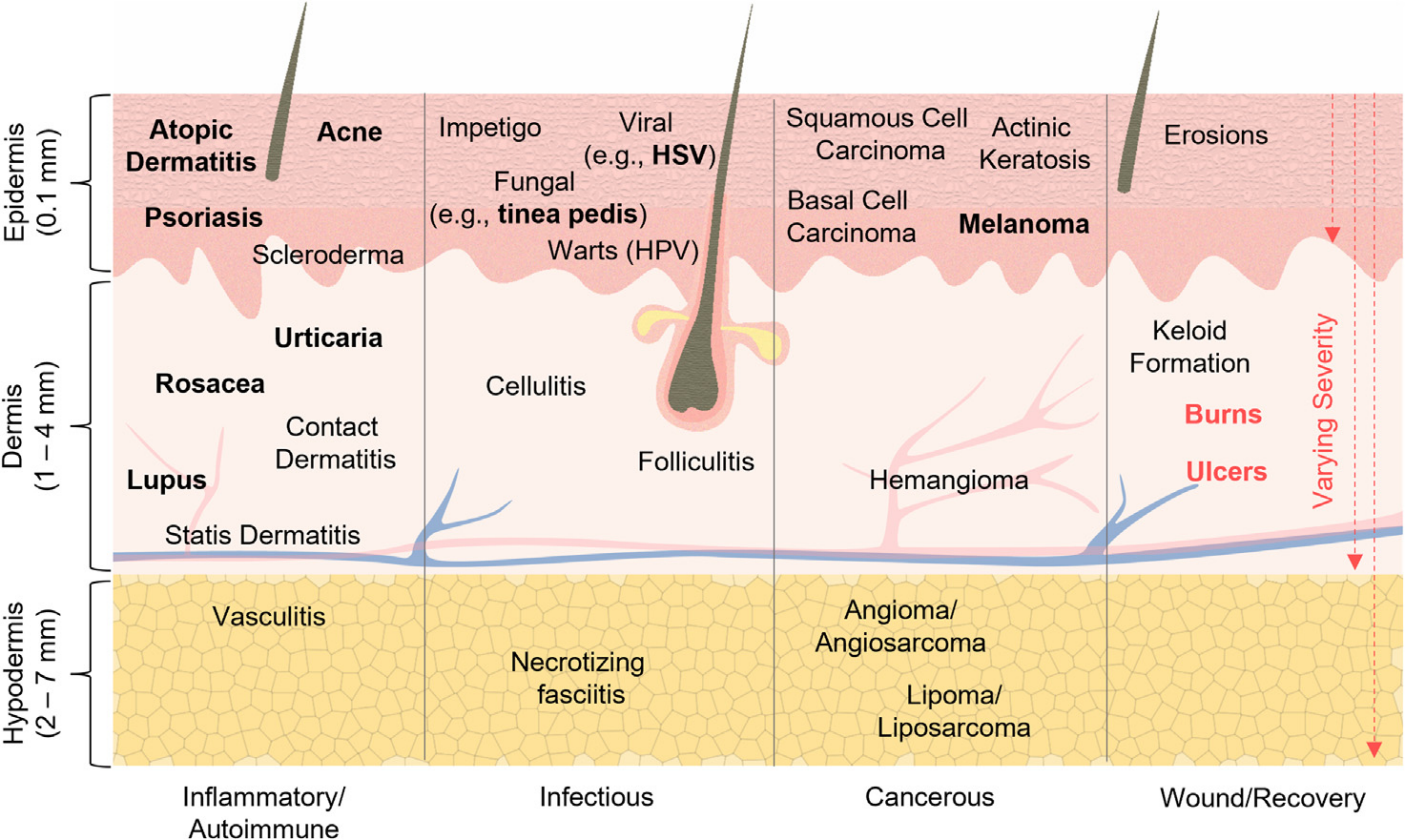
Skin and its diseases. A cross-sectional illustration of the skin and its three main layers: the epidermis, dermis, and hypodermis. The skin shields internal tissues from pathogens, harmful chemicals, physical injury, UV radiation, and water loss. The epidermis is the outermost avascular layer of the skin, followed by the dermis, a highly vascularized layer containing several important skin appendages, such as hair follicles, sebaceous glands, and sweat glands. The hypodermis is the third, vascularized and “fatty” layer of the skin, underlying the dermis. Common inflammatory/autoimmune, infectious, cancerous diseases, and wounds are listed within the layer of skin in which they originate or commonly influence. Each of these diseases/wounds impair the skin's natural functions. Red arrows in the “Wound/Recovery” panel indicate the varying severity of burns and ulcers, where greater depth indicates greater severity. Although the mechanism of each disease and layers of skin affected are diverse, visual appearance on the surface of the skin can appear similar.

Skin diseases can affect one or multiple layers depending on their pathology and severity, ultimately impairing the key functions of the skin. The underlying pathologies of skin diseases that cause physiological changes to each of these layers can be broadly classified as autoimmune/inflammatory, infectious, cancerous, and/or related to wound formation. Diseases that span the range of these categories appear in Fig. 1. Some of the most prevalent global skin disorders—acne, AD, urticaria, psoriasis, lupus, tinea pedis, melanoma, and herpes simplex virus (HSV)—are shown in bold text.^19^ To illustrate the impact of skin diseases on its properties, consider the example of AD, a common inflammatory disorder affecting 3% of adults, marked by thickening of the epidermis (lichenification) in chronic cases, and erythema with edema, formation of vesicles, and weeping in the acute stage.^20,21^ Psoriasis, on the other hand, is characterized by epidermal thickening (hyperkeratosis) forming raised, scaley plaques on the skin surface.^22^ Both of these can often have similar morphological features. As illustrated by this example, dermatological diseases are difficult to diagnose due to ambiguous symptoms—such as dryness, erythema, and edema—and require interaction with trained medical personnel for accurate assessments. Visual inspection is inherently subjective and provides little information on the affected layer of tissue. The inability to quantitatively identify and track skin disease etiology has important ramifications and motivates the need for advanced characterization techniques. Quantitative detection and monitoring of skin disease are crucial to the development of accurate diagnostics and effective therapeutics. Section II B overviews quantitative techniques for monitoring of skin health.

### B. Techniques for characterizing the properties of the skin

**FIG. 2.**
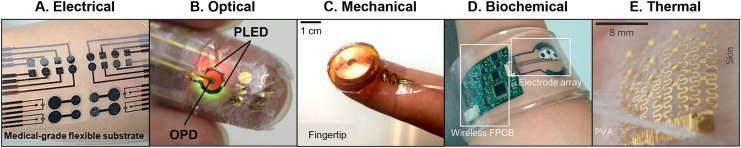
Modalities for characterizing the properties of skin. (a) Skin-interfaced device with ultrathin, functionalized hydrogel with four biosensing mechanisms, including tissue electrical impedance sensors. (b) Ultra-flexible organic optical sensor mounted on the finger for measuring photoplethysmography. (c) Electromechanical device for measurement of tissue modulus, mounted on the finger. (d) Electrochemically enhanced iontophoresis and sweat analysis device for applications in cystic fibrosis diagnosis, mounted on the arm. (e) Ultrathin, conformal array of epidermal thermal sensors for spatiotemporal mapping temperature and thermal transport properties of the skin. (a) Reproduced with permission from Lim *et al.*, Sci. Adv. **7**, eabd3716 (2021). Copyright 2021 AAAS. (b) Reproduced with permission from Yokota *et al.*, Sci. Adv. **2**, e1501856 (2016). Copyright 2016 AAAS. (c) Reproduced with permission from Song *et al.*, Nat. Biomed. Eng. **5**, 759–771 (2021). Copyright 2021 Springer Nature. (d) Reproduced with permission from Emaminejad *et al.*, Proc. Natl. Acad. Sci. U. S. A. **114**, 4625–4630 (2017). Copyright 2017 PNAS. (e) Reproduced with permission from Webb *et al.*, Nat. Mater. **12**, 938–944 (2013). Copyright 2013 Springer Nature.

Various techniques are available to assess the characteristics of the skin through measurements of its mechanical, thermal, electrical, chemical, and/or optical properties, each with different relationships to the structure, function, health, and appearance of the skin **(**Fig. 2). A brief overview of well-established modalities is useful as context for the main focus of this review on thermal methods. Measurements of electrical properties of the skin are perhaps the most common, where the skin can be approximated as a simple, parallel resistance-capacitance (R_p_-C_p_) model.^23^ The dielectric constant and electrical impedance of the skin depend on its water content and on concentrations of electrolytes.^24^ Skin temperature and skin conductance exhibit a positive monotonic relationship and can lend insight into sweat gland activity.^25^ Measurements typically involve application of an alternating voltage (*f* ∼ 20 Hz–3.5 MHz) to the skin surface through concentric ring (outer diameter ∼600 *μ*m–4 mm) or interdigitated electrodes, in direct contact or separated by a thin insulating layer.^24,26,27^ Traditional “dry” metal electrodes rely on bulk, rigid components that form contacts to the skin via applied pressure, with typically poor electrode/skin interfacial impedance and limited repeatability. “Wet” electrodes avoid these shortcomings by use of soft, conformal coatings of electrolyte, hydrogel, or ionic liquid gels to realize low interfacial impedance, improved physical contact and minimized artifacts from motion; however, drifts can arise due to changes in conductivity and/or contact impedance that follow from absorption of electrolytes/ions from the gel into the skin or vice versa, or from dehydration of the electrolyte or hydrogel [Fig. 2(a)].^28–30^ A recent work shows that thin, soft, skin-conformable, breathable classes of electrodes, sometimes referred to as “epidermal” electrodes, can form attractive approaches. Here, networks of conductive elements in random or deterministic layouts adhere to the surfaces or distribute into the bulk regions of elastomeric polymer matrices to yield conductive composites that interface to the skin via nonspecific adhesive interactions, without applied pressure and with low interfacial impedances for extended periods of wear. Such electrodes can adhere to curved regions of the body in ways that prevent constraints on natural motions or perturbations to the intrinsic rates of transepidermal water loss (Refs. 24, 26, 31, and 32).

Skin impedances at high and low *f* can provide insight into the concentration of free and bound water in the skin, across sensing depths that are tunable with measurement *f* and electrode spacing.^26,31^ Variations in electrical impedance can correlate with time dependent changes in the hydration of the skin and/or to spatial variations, of use, for example, in identifying malignant and benign cancerous tissues.^33^ The effective measurement depths are typically in a range that corresponds to the uppermost layers of the skin, the stratum corneum (∼15–20 *μ*m) and sometimes the upper epidermis (20 *μ*m–1 mm), due to the small electrode sizes/spacing and high *f* (∼100 kHz–>3.5 MHz) that are commonly used.^23,26,34^ As a result, the density of capillary beds and the influence of capillary blood flow have negligible effect. While depths can reach the millimeter-scale (0.5–2.5 mm) at low frequencies (e.g., 15 kHz) with electrode sizes in the range of centimeters (1–2.2 cm), the spatial resolution is poor.^26,34^

Optical measurements performed on the skin provide a means to evaluate the chemistry and dynamics associated with tissue water and blood,^35,36^ along with other properties that affect optical absorption and/or scattering. Illuminating the skin with single or multiple wavelengths of light and measuring the intensity and/or spectral characteristics of transmitted or reflected light can yield data relevant to the constituents of the skin, near surface tissue metabolic processes, flow properties, blood oxygenation,^37,38^ and many others. The most widely used types of such sensors determine pulse oximetry associated with blood flow within the dermis via measurements at two optical wavelengths performed using rigid finger-mounted devices. A recent work demonstrates possibilities for epidermal embodiments that rely on organic or hybrid organic/inorganic optoelectronic technologies.^39^ Specific examples include multi-wavelength sensors to assess dermal water content, optoelectronic architectures for the measurement of pulse rate/arterial blood oxygenation, and NIRS sensors to monitor tissue oxygen saturation [Fig. 2(b)].^35,36,40–43^ Such devices can be placed onto the skin at nearly any anatomical location, for measurements with reduced sensitivity to motion induced artifacts due to absence of relative motions between the devices and the surface of the skin. Additional improvements may follow from efforts to further miniaturize these devices, include dynamic digital filtering techniques, and compensate for physical motions, actively or passively. Challenges that follow from sensitivities to motions are intrinsic to all optical approaches, due to the heterogeneous structures of the skin and the relative movements of constituents of the skin during natural activities.

Measurements of the mechanical properties of the skin, such as hardness, viscosity, and tissue modulus, provide complementary information. The viscoelastic properties of the skin can be used to monitor tissue edema, tissue growth/regeneration (e.g., in wound healing), signs of aging, and reactions to exogenous substances.^44–48^ Nanoindentation, mechanical suction, torsion, and ultrasound dynamic elastography represent established techniques. These approaches are typically applied to small, approximately planar regions of the body for spot measurements, with limited ability for continuous data collection.^47,49–53^ The most recent technologies adopt wearable designs, using thin flexible sheets of piezoelectric or electromagnetic actuators and sensors [Fig 2(c)]. Oscillating forces imparted to the skin by these devices yield displacements that can be sensed to determine intrinsic modulus values. The effective depths of the measurements can extend from the upper regions of the epidermis to the dermis and below, depending on the device configuration. The results provide unique insight for diagnosis/monitoring of cutaneous disorders (e.g., psoriasis, ulcers), muscular activity, and signs of tissue ageing for applications in cosmetics.^47,48,54^

Measurements of biochemical characteristics of the skin can complement those of biophysical parameters discussed in preceding sections. Here, skin pH and the concentrations of biomarkers in sweat are of growing interest^55–59^ due to their relationship to physiological health, hydration status, and electrolyte balance.^55,57,58^ For example, chloride concentrations in sweat serve as clinical indicators for detecting and monitoring cystic fibrosis **[**Fig. 2(d)].^55,59,60^ Some studies suggest that glucose concentrations in sweat correspond approximately to those in blood, of relevance to screening and tracking of conditions related to diabetes. Other sweat biomarkers can lend insight into nutritional health (e.g., electrolytes such as Na^+^ and K^+^) and metabolic activity (ethanol, uric acid, and lactate), with good correlations to concentrations in blood for certain cases (similar to glucose). Similarly, the presence of substances, such as heavy metals (e.g., Cu, Zn, Hg, and Cd) and drugs (e.g., methylxanthine and nicotine), in sweat can be important indicators of health. Commercial devices exist for using sweat alcohol concentrations as approximate measures of alcohol levels in the blood.^61–64^ Sensing in these various scenarios involves electrochemical, colorimetric, or fluorometric methods applied to sweat at the surface of the skin or to microliter volumes captured in skin-compliant microfluidic reservoirs and channels.^58^ Water can also escape from the skin in the form of vapor due to permeation through the stratum corneum. Known as transepidermal water loss (TEWL), this process is widely used to characterize the efficacy of the barrier function of the stratum corneum.^65^ TEWL, with units of g m^−2^ h^−1^, is inferred from measurements of water vapor flux using clinical instrumentation. While portable meters exist to measure skin TEWL, these instruments are expensive and rigid, preventing continuous measurements.^66,67^

Skin pH is also relevant to barrier function, as an acidic stratum corneum (pH ∼ 4–6) is responsible for maintaining the mechanical integrity and the homeostasis of the epidermal barrier.^68–70^ Inflammatory diseases, such as atopic dermatitis, contact dermatitis, ichthyosis, rosacea, and acne, as well as dry and aged skin all exhibit a relative increase in pH compared to that of healthy skin.^71^ Glass planar electrode instruments are commonly used to measure skin surface pH, but require precise and frequent calibrations in standard buffers for accurate measurements.^72^

The final type of skin measurement summarized in this section involves thermal properties, the primary focus of the remainder of this article. Spatiotemporal distributions of temperature at the surface of the skin, along with thermal transport properties both in the near surface regions and at underlying depths, represent additional, complementary sets of characteristics that can yield important insight. Recent advances in devices and approaches for rapid, accurate measurements of thermal characteristics form the basis of growing interest in this area.^31,73–80^ Temperature measurements performed on the surface of the skin are important as a surrogate for, or extrapolation of, core body temperature, one of the six clinical vital signs used to assess overall health status.^81–83^ Measurements of local skin temperature can also be used to assess dermatological disease severity, presence of melanoma, and other conditions.^84,85^ Body temperature is also instrumental in monitoring circadian rhythms and sleep quality.^46,86^ Within the last decade, development of high-precision, high-resolution (∼0.03 °C) temperature sensors with fast response times (5–30 ms), with designs that do not disrupt the skin's natural physiological processes, such as TEWL, enable observations of physiological phenomena, such as changes in cognitive state and microcirculation to the skin [Fig. 2(e)].^76,80^ Aside from surface temperature, the thermal transport properties of the skin, such as the thermal conductivity *k* and thermal diffusivity *α*, relate directly to changes in subsurface microvascular/macrovascular blood flow and tissue water content. Some of the most advanced devices for measuring thermal transport are reusable, nondestructive, tunable (to sense tissue properties at varying depths, ∼0.5–6 mm), multimodal, and applicable to nearly any region of the body.^76,78,79,87,88^ The ability to create arrays of thermal sensors with small lateral dimensions (∼0.25 mm^2^ or smaller) and with relatively large measurement depths (∼0.5–2 mm) allows for high-resolution spatial mapping of the thermal transport properties of the skin and determination of degree of anisotropy of microvascular and macrovascular blood flow in superficial veins and arteries.^75,76^ Reported investigations on subjects with neurological disorders and common dermatological diseases highlight the relevance and applicability of this field of research to real-world clinical cases.^79,87,88^

### C. Thermal properties of the skin

The two main thermophysical properties associated with heat transport are the ability of a material to store and to transfer heat.^89^ The former is given by its heat capacity *c*, where the temperature increase d*T* caused by the addition of heat d*Q* to a thermally isolated material of mass *m* is given by^89^

dQ=mcdT. (1)

Equation (1) is a statement of the first law of thermodynamics. We ignore the distinction between heat capacity at constant pressure (*c*_p_) and constant volume (*c*_v_) as solids and liquids—the relevant constituents of the skin—can be considered “incompressible” (material volume is constant for variations in pressure, so *c*_p_ = *c*_v_ = *c*).^89,108^ In simple terms, the heat capacity of a material describes the amount of heat required to raise the temperature of a sample of mass *m* by one unit of temperature. For skin modeled as a mixture of spherical droplets of water embedded within a matrix of tissue, the product of tissue density and *c*_p_ is linear with respect to the volume fraction of water in skin.^31^ Data show that this model of the skin provides a reasonable approximation through studies of *in vitro* porcine skin models.^31^ To aid theoretical discussions, a list of key variables used in this paper appears in Table II.

**TABLE II.** List of frequently used variables in the paper.

Variable	Description	Units	Variable	Description	Units
*b*	Line heater half-width	*μ*m	*s*	Heater-sensor separation distance	mm
*c*	Heat capacity	kJ kg^−1^ K^−1^	*T*	Temperature	°C
*d*	Layer thickness	mm	*t*	time	s
*f*	Frequency	Hz	ToF	Time of flight	s
*H*	Heat transfer coefficient	W m^−2^ K^−1^	*v*	Flow velocity	m s^−1^
*h*	Skin or thermal penetration depth	mm	*V*	Voltage	V
*i, I*	Alternating/constant current	mA	*w*	Blood perfusion	s^−1^
*k*	Thermal conductivity	W m^−1^ K^−1^	*α*	Thermal diffusivity	mm^2^ s^−1^
*L*	Line heater length	mm	*β*	Temperature coefficient of resistance	% ·°C
*m*	Mass	g	*ε*	Emissivity	⋯
*P*	Electrical power	mW	*λ*	Wavelength	nm or *μ*m
*Q*	Heat	J	*ρ*	Density	kg m^−3^
*q*	Heating power per unit area	mW · mm^−2^	τ	Dimensionless time	⋯
$\dot{q}$	Heat transfer rate per unit area	W · m^−2^	*φ*	Water content	%
*R*	Resistance	Ω	ϕ	Phase	° or rad
*r*	Radius	mm	*ω*	Angular frequency	rad · s^−1^

Heat transport through conduction is the primary method of heat transport in solid bodies like skin and occurs predominantly through molecular interactions. Thermal conduction is described through Fourier's Law as follows:

$$\vec{q}=-k\vec{\nabla T},$$ (2)

where *q* is the heat flux density in W m^−2^, and ∇*T* is the local temperature gradient. The coefficient ***k*** is a second-order tensor and describes the thermal conductivity, which can vary with temperature and relative position within the material.^108^ This review largely considers skin and/or its individual layers as isotropic and homogeneous, with values of ***k*** that have negligible dependence on temperature (without considering the effects of perfusion), consistent with *ex vivo* studies of tissue models.^93^ Thus, the tensor ***k*** reduces to a scalar, *k*. Thermal conductivity describes both transient and steady-state conduction.^89^ For human skin, values of *k* of the epidermis and dermis lie in between the literature reference for dry skin and for blood and water, as illustrated in Table III. The value of *k* of the hypodermis is lower than that of the epidermis/dermis, as this layer is primarily composed of fatty tissue. Dermatological diseases result in changes of the water content, vascular blood flow, barrier properties, and composition of one or more layers of the skin (e.g., induce thickening of the epidermis). In turn, changes in the water content and blood flow can be reflected in measured values of the thermal transport properties of the skin.

**TABLE III.** The thermal properties of the skin.

	*k* (W/m K)	*α* (mm^2^/s)	*ρ* (kg/m^3^)	*v* (ml/min)	*w* (s^−1^)
Blood	0.52^90^	0.15^91^	1030–1060^92,93^	0.1–600^14,94^	⋯
Water	0.6^95,96^	0.15^96^	997^97^	>3 × 10^−3^ (ISF^*^)^98,99^	⋯
Epidermis	0.2 (dry)–0.6 (hydrated)^100,101^	0.028–0.18^102^	1190^92^	⋯	0 (Ref. 103)
Dermis	0.2 (dry, unperfused)–0.6 (hydrated, perfused)^91,104–106^	0.11–0.18^92^	1116^92^	⋯	1.25 × 10^−3^ (Ref. 103)
Hypodermis	0.19^92,103^	0.05–0.2^92^	971^92^	⋯	2.5 × 10^−3^ (Ref. 107)

Typically, both heat conduction and storage occur simultaneously.^89^ The transient heat conduction equation describes the “competing” nature of heat conduction and storage as follows:

$$\frac{\partial T}{\partial t}=\alpha \nabla^{2}T.$$ (3)

Equation (3) applies for an isotropic, incompressible material. The diffusivity, *α*, is the ratio of the thermal conductivity to the product of the tissue density *ρ* and *c*,

$$\alpha =\frac{k}{\rho c}.$$ (4)

Physically, *α* is the ratio of heat conducted to heat stored within a material and denotes the speed of heat propagation through a material subject to a temperature gradient.^89^ Unlike *k* which describes both transient and steady-state heat conduction, *α* is only defined during transient heat conduction. Like *k*, *α* of human skin lies in between values of dry skin, blood, and water, but it has a non-monotonic relationship with respect to blood and water content.^31^

Convection, another mechanism of heat transport, occurs via the movement of a fluid away from a surface. Convection can be natural, due to local variations in fluid temperature, or forced by an external agent, such as a pump.^109^ For applications in skin, convection of blood through veins/arteries is “forced” by the heart. The Newton rate equation, or the rate equation for convective heat transfer, defines the heat transfer coefficient *h* as follows:

$$\dot{q}=h\Delta T,$$ (5)

where $\dot{q}$ is the rate of convective heat transfer per unit area normal to the direction of heat flow in J/s/m^2^ (W/m^2^), and Δ*T* is the difference in temperature between the surface and the fluid. The heat transfer coefficient denotes the rate of heat transfer between a solid surface and a moving fluid. The skin cannot be treated as a pure solid body, as it contains fluids, such as blood and water, which can dissipate heat through convection. The rate of perfusion, *w*, or the blood flow velocity per unit volume of tissue, can be derived from thermal transport measurements by detecting convective heat losses, which will be described in Sec. V. The avascular epidermis naturally has *w* = 0 while the dermis and hypodermis are both vascularized and, therefore, have finite *w* > 0; the hypodermis experiences more blood flow than the dermis. On the other hand, the directional flow rate of blood (*v*) in the skin (e.g., through veins) can vary from 10 ml/min (metacarpal vein) to 500 ml/min (portal veins) and can change based on cardiac output.^110^ The flow of water in the skin (e.g., interstitial fluid) is much lower, ∼0.2 ml/min, and is typically isotropic.^111^ Section V describes experimental methodologies to distinguish between the isotropic and anisotropic flows.

Radiative heat loss occurs through electromagnetic radiation between two bodies. The Stefan–Boltzmann law describes the rate of energy emission from the skin to the environment as follows:^109,112,113^

$$\dot{q}=A\sigma \varepsilon_{\mathrm{skin}}\varepsilon_{\mathrm{air}}(\;T_{\mathrm{skin}}^{4}-T_{\mathrm{air}}^{4}),$$ (6)

where $\dot{q}$ is the rate of radiant energy emitted per unit area of the exposed skin surface; *T*_skin_ and *T*_air_ are absolute temperature of the skin and air, respectively; *ε*_skin_ and *ε*_air_ are the emissivity of the skin and air, respectively; and *σ* is the Stefan–Boltzmann constant. Equation (6) illustrates that radiative effects are dominant at large differences in skin/air temperature and are negligible for the physiologically relevant temperatures (∼310 K or 37 °C) with respect to room temperature (∼298 K or 25 °C).

Skin temperature arises from thermal energy balance within the body, an additive combination of the body's metabolic heat production and the heat lost/gained by various heat transfer processes,^114^

$$\Delta E=M-(W-Q_{\mathrm{conv}}+Q_{\mathrm{cond}}+Q_{\mathrm{rad}}+Q_{\mathrm{evap}}+Q_{\mathrm{resp}}),$$ (7)

where Δ*E* is the rate of energy storage in the body, *M* is body's metabolic energy produced, *W* is the external work performed by the body, and *Q* represents heat gain/loss, with various subscripts indicating the different heat transfer mechanisms (conv = convection, cond = conduction, rad = radiation, evap = evaporation and resp = respiration). Pennes' bioheat transfer equation, derived from this energy balance equation, can be used to predict temperature distributions inside the skin and other tissues:^114,115^

$$\rho c\frac{\partial T}{\partial t}=\nabla \cdot k\nabla T+\rho_{\mathrm{bl}}c_{\mathrm{bl}}w\left(T{}_{a}-T\right)+M,$$ (8)

where *T*_a_ represents the arterial blood temperature, and *T* is the surrounding tissue temperature. This model describes the effect of metabolism and blood perfusion on the energy balance within the tissue. While Eq. (8) relies on many simplifying assumptions (e.g., isotropic, homogeneous tissue properties), advanced models can be used to capture additional details.^116^

## III. TECHNIQUES FOR SPATIOTEMPORAL MAPPING ON THE SURFACE OF THE SKIN

Simple, accurate measurements of skin surface temperature, particularly performed with wearable devices, are of growing interest given their physiological significance. Chung *et al.* demonstrate the use of compact, wearable devices that interface gently and noninvasively with the skin of neonates for the measurement of the full suite of vital signs in the intensive care unit (ICU), including clinical grade thermometry on the skin of as a surrogate for measuring core body temperature.^81,82^ Wearable temperature sensors can also be used to monitor mental and physical activity,^76^ emotions,^117^ sleep cycles, and menstrual cycles.^86,118,119^ Furthermore, skin temperature has applications in monitoring of skin injury, such as wound healing, viability of skin flaps, and ulcer formation (e.g., diabetic ulcers, bed sores), and the onset of disease, such as breast cancer.^46,120–122^

Critical criteria for characterizing wearable temperature sensors are temperature resolution, response time (low thermal mass), and hysteresis-free behavior, each of which can be addressed with appropriate device designs and materials selections. In addition, intimate thermal contact with the skin is vital for all of these types of measurements.^123^ To minimize thermal contact resistance, the devices must conform to the nonplanar, rough surface of the skin without air gaps. From an analysis of the contact mechanics, this scenario occurs when^124^

$$\frac{\pi {\bar{E}}_{\mathrm{skin}}h_{\mathrm{rough}}^{2}}{\gamma \lambda_{\mathrm{rough}}}<16+\frac{{\bar{E}}_{\mathrm{skin}}\lambda_{\mathrm{rough}}^{3}}{\pi^{3}{\bar{EI}}_{\mathrm{device}}},$$ (9)

where ${\bar{E}}_{\mathrm{skin}}$ is the plane-strain modulus of the skin; *h*_rough_ and *λ*_rough_ are the amplitude and wavelength of the roughness of the surface of the skin, respectively; ${\bar{EI}}_{\mathrm{device}}$ is the bending stiffness of the device; and *γ* is the work of adhesion between the surface of the device and the skin. Equation (9) assumes that *λ*_rough_ ∼ 7*h*_rough_, as shown in experimental measurements of skin roughness.^124^ This scaling law demonstrates that devices with low effective modulus and bending stiffness (i.e., soft and thin), and strong adhesion to skin promote conformal contact. Establishing such contact becomes increasingly difficult as the roughness and modulus of the skin increases. The amplitude of features in the skin like wrinkles, creases, and pits are 15–100 *μ*m and their sizes are 40–1000 *μ*m, and the Young's modulus of the skin is 140–600 kPa for the epidermis and 2–80 kPa for the dermis; in practice, for typical filamentary serpentine epidermal devices (silicone/PI/Au) reported in Ref. 124 with *d*_silicone_ = 30, *d*_PI_ = 0.3, and *d*_gold_ = 0.2 *μ*m, *γ* = 0.16 Ν/m, and ${\bar{EI}}_{\mathrm{device}}$ = 0.27 × 10^−0^ N m, devices have conformal contact with the skin when *h*_rough_ ≤56 *μ*m.^124,125^

### A. Contact-based techniques for temperature sensing

#### 1. Resistance temperature detectors

Sensing mechanisms typically involve electrical or optical means. The former exploit resistive temperature detectors (RTDs), positive temperature coefficient (PTC) or negative temperature coefficient (NTC) thermistors, or thermocouples.^76,79,126–130^ RTDs for measurements of skin temperature, often constructed using inert, biocompatible metals, such as Au or Pt, exhibit a change in electrical resistance with temperature. For most metals, at small values of Δ*T*,

$$\Delta T=\frac{\Delta R}{R_{0}\beta },$$ (10)

where $\Delta R=R-R{}_{0}$, *R*_0_ is the nominal resistance and *β* is the temperature coefficient of resistance (TCR) of the metal. Values of *β* and resistivity for copper, gold, and platinum are *β* = 43 × 10^−4^/K, resistivity = 1.67 × 10^−8^ Ω m, *β* = 39 × 10^−4^/K, resistivity = 2.30 × 10^−8^ Ω m), and *β* = 39.2 × 10^−4^/K, resistivity = 10.6 × 10^−8^ Ω m, respectively.^131,132^ While the highly linear relationship between *R* and *T* for RTDs is appealing, these small values of TCR typically demand the use of amplification electronics for reliable measurements.^76,79^

**FIG. 3.**
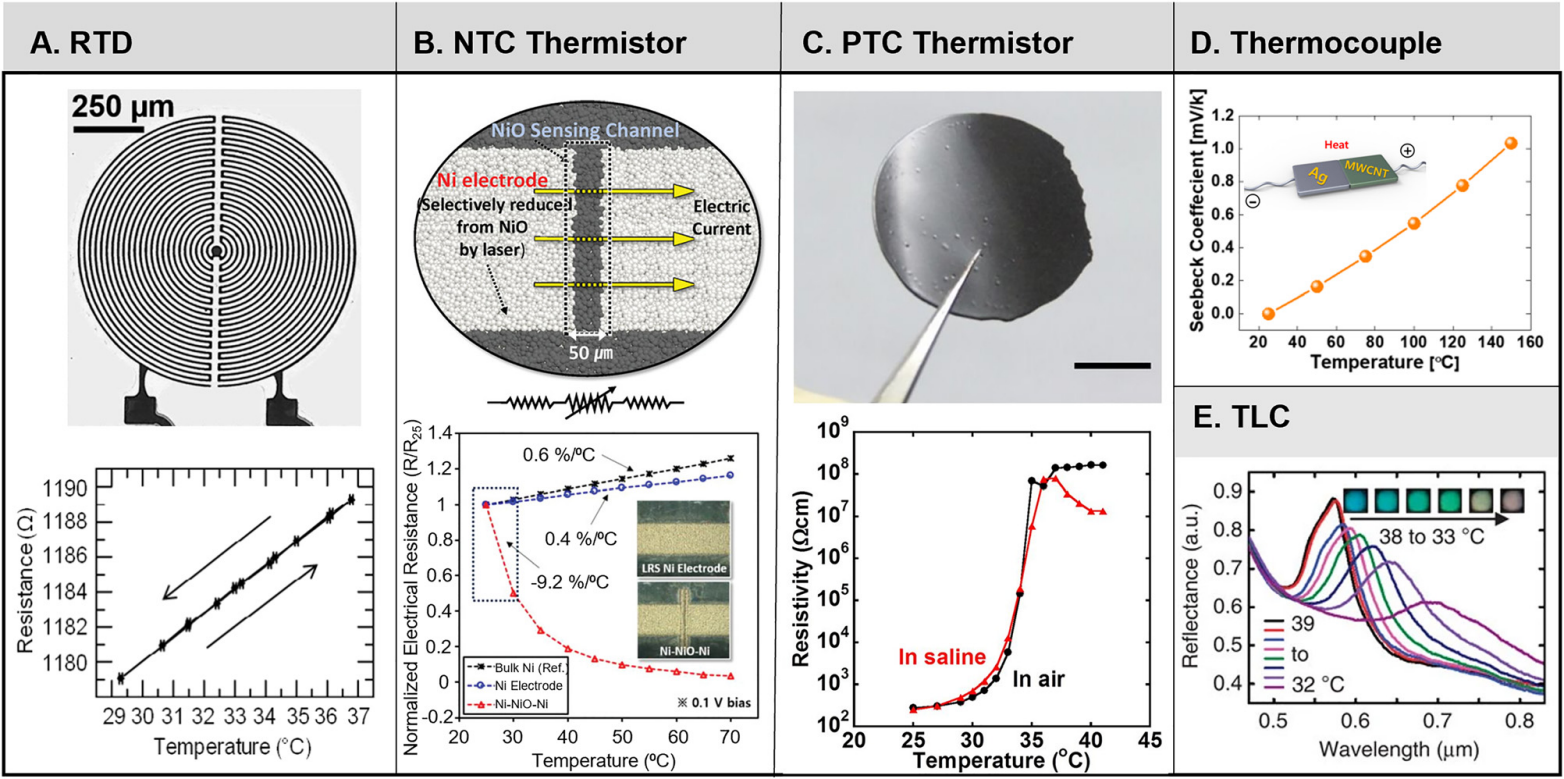
Overview of wearable temperature sensors. (a) Optical image of a resistance temperature detector (RTD) made of a winding trace of 100-nm-thick Au (top) and the resistance response of a typical thin film Au-based RTD with respect to temperature (bottom). (b) Schematic image of a flexible, sintered NiO negative temperature coefficient (NTC) thermistor (top) and resistance vs temperature curves for the completed Ni–NiO–Ni sensor and pure Ni electrodes (bottom). (c) Optical image of a positive temperature coefficient (PTC) thermistor composed of graphite and semicrystalline acrylate copolymers (top) and corresponding resistivity vs temperature response of the sensor in air and saline environments. (d) Seebeck voltage vs temperature of a flexible silver nanoparticle/multiwalled carbon nanotube (MWCNT) thermocouple for e-skin applications. A schematic image of the sensor is in the inset. (e) Reflectance measurement as a function of temperature for a single pixel of an array of thermochromic liquid crystals (TLC) mounted on a soft elastomer substrate. The corresponding images of the single TLC pixel is in the inset. (a) (top) Reproduced with permission from Crawford *et al.*, Extreme Mech. Lett. 22, 27–35 (2018). Copyright 2018 Authors, licensed under a Creative Commons Attribution (CC BY) license. (bottom) Reproduced with permission from Webb e*t al.*, Nat. Mater. **12**, 938–944 (2013). Copyright 2013 Springer Nature. (b) Reproduced with permission from Shin *et al.*, Adv. Mater. **32**, 1905527 (2019). Copyright 2019 Authors, licensed under a Creative Commons Attribution (CC BY) license. (c) Reproduced with permission from Yokota *et al.*, Proc. Natl. Acad. Sci. U. S. A. **112**, 14533–14538 (2015). Copyright 2015 PNAS. (d) Reproduced with permission from Jung *et al.*, Flexible Printed Electron. **5**, 025003 (2020). Copyright 2020 IOP Publishing. (e) Reproduced with permission from Gao *et al.*, Nat. Commun. **5**, 4938 (2014). Copyright 2014 Springer Nature.

Soft, ultrathin devices, sometimes referred to as “epidermal” devices, represent the most attractive construction of RTDs (and other temperature sensing mechanisms),^76,125^ where winding traces (∼20 *μ*m wide) of thin films (∼50–100 nm thick) of an inert metal, such as Au, serve as the heaters and sensors [Fig. 3(a)].^73,76^ Encapsulation above and below using biocompatible polymers (e.g., polyimide (PI), with thicknesses *d*_PI_ between ∼1 and 6 *μ*m) form chemically resistant, electrically insulating, mechanically robust (∼2.8 GPa elastic modulus) protective layers that isolate these traces from the surroundings (e.g., from environmental humidity, water/fluids). With appropriate choices in thickness, these layers can also place the metal features at the neutral mechanical plane to enhance the bendability of the resulting structure.^76,124^ An elastomer backing material (typically poly(dimethyl)siloxane (PDMS), ∼50–100 *μ*m thick) allows repeated application and removal of the device from the skin, as a soft, conformal physical interface.^76,78^ Such devices have small thermal mass (7.2 mJ cm^−2^ K^−1^), fast response times (4–13 ms), and high levels of elastic stretchability (∼10%, can be > 50%).^74,76^ The polymer encapsulants and their thicknesses (*d*_PI_, *d*_silicone_), furthermore, are key in defining the thermal mass and ultimately the response times of the RTD devices. Typical silicone elastomer backing materials increase the thermal mass per unit area by 48 × over that of the PI/metal/PI structures alone (150 *μ*J cm^−2^ K^−1^).^76^ The response time for a device structure with *d*_PI_ = 3.6 *μ*m, *t*_silicone_ = 50 *μ*m is 4.2 ms. Increasing the *d*_PI_ to 6 *μ*m leads to a threefold increase in response time.^76^ Ultrathin backing layers, in perforated or non-perforated forms, can be highly gas/water vapor permeable, thereby accommodating natural physiological processes like TEWL (i.e., skin hydration) without perturbing the natural temperature of the surface of the skin across most scenarios of interest.^76^ Commercial, surface-mount components (SMD/SMT) (∼0.9 × 0.9 mm^2^) can also serve as heating and sensing elements, but accurate measurements require calibration to account for their heat capacities.^79,87^ Thin metal films are attractive for their relatively small heat capacities (e.g., gold, *c* = 0.13 kJ kg^−1^ K^−1^) compared to those of ceramics (e.g., silicon carbide, *c* = 0.67 kJ kg^−1^ K^−1^) and for their ease of patterning into small features and/or desired geometries.^73^

#### 2. Thermistors

NTC thermistors (as well as PTC thermistors), in contrast with RTDs, exhibit much larger variations in resistance as a function of temperature, as given by^133^

$$R_{T}=R_{\infty }e^{B/T},$$ (11)

where *R*_T_ is the resistance at temperature *T*, *R*_∞_ is the reference resistance of the thermistor, and *B* is the “B-value,” also known as the “thermistor value.” While NTC/PTC thermistor responses are typically nonlinear with respect to *T*, for small ranges, the dependence can be approximated as linear with a corresponding value of TCR. Conventional thermistors are typically ceramic materials based on sintered semiconducting oxides (e.g., transition metal oxides like NiO, FeO, CoO, and MnO). Polymers/polymer composites are increasingly popular, due to their ease of processing and their mechanical flexibility.^134,135^ At *T* ∼ 25 °C, the TCR for the flexible, sintered 30-*μ*m-thick NiO NTC thermistor fabricated on a thin PET substrate (25 *μ*m) from Ref. 133 is −9.2% C^−1^, much larger than values for RTDs [Fig. 3(b)].^133^ The thin PET substrate enables a response time of < 50 ms for this sensor structure. These flexible NiO NTCs can be used for applications in artificial skin (touch sensitivity to heat/cold) or as monitors of changes in the temperature of the skin associated with activity (e.g., cycling). Additional possibilities are in tracking respiratory rate when placed in physiologically relevant locations, such as under the nasal cavity.^133^ Sang *et al.* demonstrate Au-doped silicon nanomembrane (SiNM) NTC thermistors with relatively fast response times (<9 ms due to ∼300 nm Au-doped SiNM thickness and ∼1.5 *μ*m PI encapsulation on either side) compared to materials which exhibit phase-change mechanisms (i.e., polymer-based thermistors) and a TCR 22 × higher than that of thin film RTDs.^136^ The magnitude of the TCR of the Au-doped SiNM increases with decreasing doping temperature and dopant concentration^136^ according to

$$\mathrm{TCR}=-\frac{E_{D}-E_{V}-K_{B}Tln\left(\frac{N_{a}}{2\left(N_{\mathrm{Au}}-N_{a}\right)}\right)}{T^{2}},$$ (12)

where *E*_D_ is the donor level energy of Au, *E*_V_ is the highest energy level of the valence band, *K*_B_ is Boltzmann's constant, *N*_a_ is the concentration of acceptors (Boron) in Si, and *N*_Au_ is the Au dopant concentration. Experiments demonstrate improved signal to noise ratios (SNR) in body-worn applications, where the Au-doped SiNM sensor can detect appreciable changes in signal due to temperature fluctuations on the body during alternating periods of rest and exercise. By comparison, conventional metal-based RTDs exhibit very low percentage changes in resistance in similar conditions.^136^

Yokota *et al.* report classes of flexible, printable PTC thermistors comprised of a composite of graphite (2–3 *μ*m diameter particles) and semicrystalline acrylate copolymers with resistance changes of six orders of magnitude over temperature changes of only 4 °C, response times of < 100 ms, and temperature sensitivities of 20 mK [Fig. 3(c)]. These properties address the challenge of developing PTC thermistors with sensitivity at physiological temperatures.^135^ The “PTC” mechanism arises from an increase in specific volume of the composite as a function of temperature through the melting point (*T*_M_ = 36 °C). The density of the polymer composite is reported to decrease by ∼5% above *T*_M_ as the polymer undergoes a phase transition from semicrystalline to amorphous.^135^ For applications on skin, the response must be immune to bending and thermal cycling. Characterization results show that the devices are unaffected by bending radii to values as small as < 700 *μ*m. Negligible changes occur over 200 cycles of bending to radii of 1 mm and 1800 thermal cycles from 29.8 to 37 °C. These excellent performance characteristics follow, at least partly, from the use of a “lateral-type” structure where the PTC film lies between two neighboring electrodes on the x–y plane, instead of between electrode films above and below in the vertical z-direction.^135^

#### 3. Thermocouples

Thermocouples operate on the principle of the thermoelectric effect, where an electromotive force (EMF) results from the junction between two dissimilar conductors upon heating.^137^ The Seebeck coefficient *S*, analogous to TCR, describes the induced voltage change *V* with respect to change in temperature:

$$S=\frac{V}{\Delta T}.$$ (13)

The two constituent materials for thermocouples must have a sufficiently large difference in electronic work function, *Φ*. The Seebeck coefficient for two commonly used metals in thermocouples—copper and constantan—is 40 *μ*V K^−1^. Reference 126 reports a flexible thermocouple for use in electronic-skin applications that combines silver nanoparticles (*Φ* ∼ 4.2 eV) and multiwalled carbon nanotubes (*Φ* ∼ 4.6 eV) inkjet-printed onto a flexible indium-tin-oxide (ITO)-coated PET substrate. For this system, *S* = 8 mV K^−1^ for 25–150 °C [Fig. 3(d)].^126^ Thermocouples are attractive for certain applications, but they can undergo baseline drift in wearable devices due to the difficulty in maintaining a precise reference temperature at one side of the metal junction.^134,138^

#### 4. Thermochromic liquid crystals

Apart from traditional temperature sensing mechanisms (RTDs, thermistors, thermocouples), colorimetric temperature sensing systems comprising pixelated thermochromic liquid crystal (TLC) arrays on thin, soft elastomeric supports represent an additional option with demonstrated utility in the thermal characterization of the skin.^80^ Gao *et al.* report a patterned TLC ink (∼25 *μ*m thickness, 250 or 500 *μ*m diameter and spacing), on black poly(dimethyl) siloxane (PDMS) with < 30 ms response times [Fig. 3(e)].^80^ Hue and saturation extracted from digital camera photos using computer vision algorithms quantitatively relate the colorimetric response to temperature.

### B. Noncontact techniques for temperature mapping

Infrared thermography is the most common, non-contact optical technique used for noninvasive temperature mapping of the skin surface. The sensitivity of IR cameras is 0.01–0.05 °C, and spatial resolution can be < 2 mm over large fields of view: 200 × 200 mm^2^–500 × 500 mm^2^.^139^ The images provide information about local cutaneous perfusion, metabolism, and environmental temperature.^122^ The temperature sensed by the IR camera (*T*_sensed_) is given by^122^

$$T_{\mathrm{sensed}}=\left[\frac{\varepsilon T_{\mathrm{skin}}^{4}+\left(1-\varepsilon \right)T_{\infty }^{4}}{T_{\mathrm{skin}}^{4}}\right],$$ (14)

where *ε* is the emissivity of the skin, *T*_skin_ is the true skin temperature, and *T*_∞_ is the ambient room temperature. For radiative emission with wavelengths in the range of 3–14 *μ*m, *ε* is independent of pigment, and for skin at 303 K and a room temperature of 293 K, a variation in *ε* of ± 0.01 (from the literature on *in vivo* and *in vitro* subjects) corresponds to an apparent variation of temperature of ± 0.01 °C.^122,140^ Infrared thermography requires instrument calibration, and measurements often rely on symmetric sites on the body as points of reference. Surface curvature and viewing angle also can lead to measurement errors, thereby frustrating studies on complex geometries of the body and requiring subjects to remain stationary during image recording.^139^

### C. Applications of temperature sensing of the skin surface

**FIG. 4.**
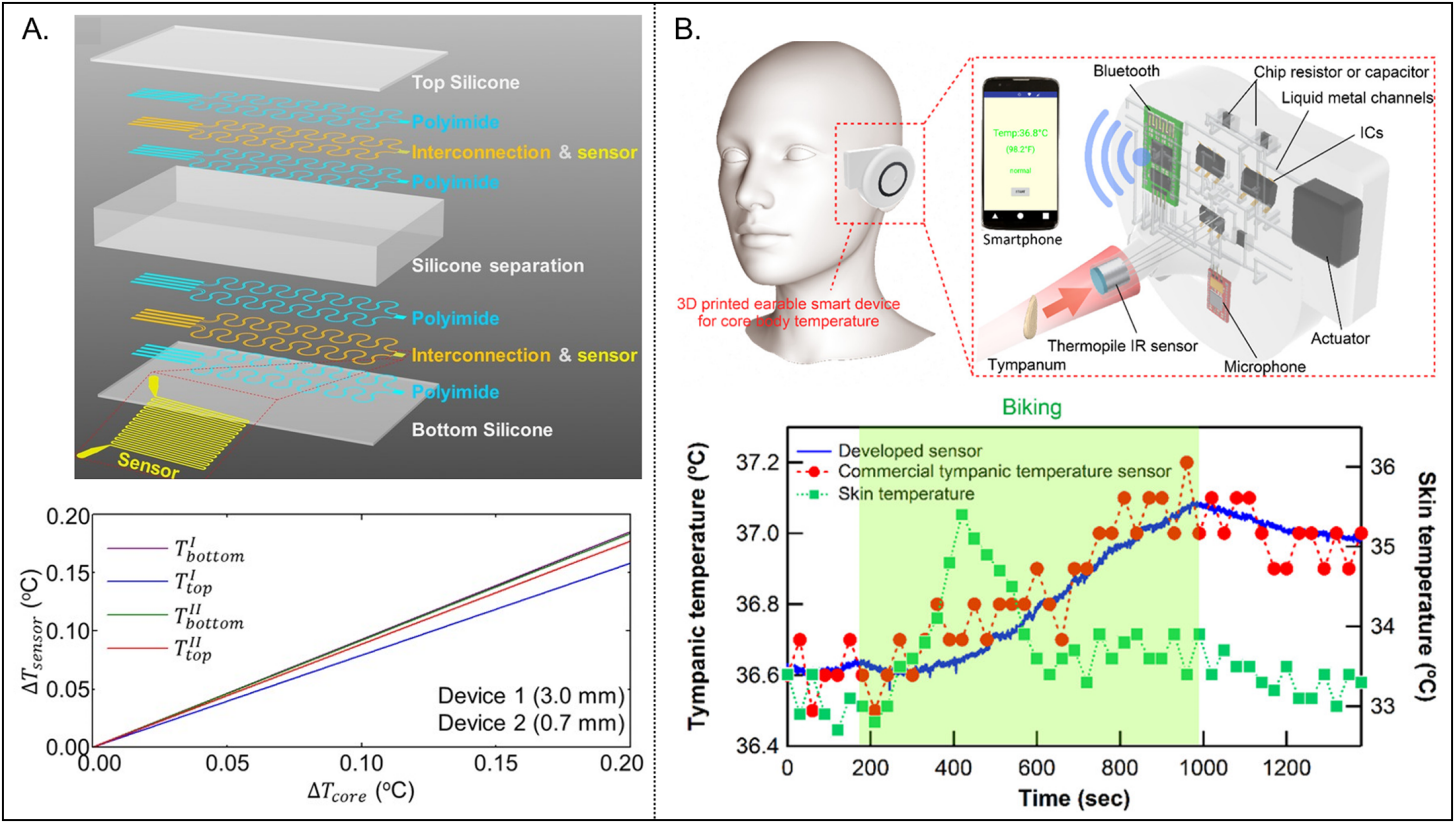
Wearable temperature sensors as proxies for core body temperature. (a) Layer diagram of a soft, flexible, multi-layered temperature sensing platform for measurements of core body temperature (top) and FEA simulation of the measured temperature of two representative devices as a function of core body temperature (bottom). (b) Schematic image of a 3D printed “earable” smart device for measurement of core body temperature through the surface temperature of the tympanum (top) and developed sensor, commercial tympanic temperature sensor, and skin temperature data collected on a subject during exercise (bottom). (a) Reproduced with permission from Zhang *et al.* Adv. Healthcare Mater. **5**, 1 (2015). Copyright 2015 Wiley VcH. (b) Reproduced with permission from Ota *et al.*, ACS Sens. **2**, 990–997 (2017). Copyright 2017 ACS.

Simple, point measurements, or spatiotemporal mapping of skin surface temperature (*T*_skin_) can be valuable in many applications. Table IV highlights a few key examples. Epidermal or skin-mounted temperature sensors have garnered interest, at the most basic level, as a surrogate for measurements of core body temperature (*T*_core_). Zhang *et al.* demonstrate differential measurements from RTD sensors at different distances away from the skin, separated by layers of silicone [Fig. 4(a)].^77^ Through an approximated 1D heat conduction analysis, theoretical analysis indicates that the temperatures at these distances depend on *T*_core_, the thermal properties of the underlying tissue, and the ambient conditions, such as temperature and air convection. Extraction of the unknowns (i.e., *T*_core_, *t*_tissue_/*k*_tissue_ where *t*_tissue_ is tissue thickness, and convection coefficient *h*) occurs through numerical error minimization analysis. Uncertainties that result from this fitting process can be minimized by increasing the total number of sensors.^77^ The published results include the impact of various environmental conditions, including those that involve air convection, on differential measurements to determine of *T*_core_. A disadvantage of this approach is the relatively long response times (∼474 to ∼1030 s) that follow from the thicknesses of the separating silicone layers in this device architecture.^77^

**TABLE IV.** Representative applications of temperature sensing of the skin surface.

Figure	Reference	Application	Sensor type	Sensing location	Sensing mode	Key details
Figure 4(a)	77	*T*_core_ sensing	Dual RTD (thin film Au), soft flexible sensors with polyimide and silicone encapsulation	Epidermal	Differential single point Single time	• Dual sensors separated from skin at different distances enable correction for variations in ambient temperature, skin properties, and heat loss from air convection Long response time (∼474–1030 s)
Figure 4(b)	141	*T*_core_ sensing	Thermopile IR sensor, thermistor	Ear/tympanum	Differential single point Continuous time	• Tympanum is less impacted by environmental variations, resulting in more accurate reading of *T*_core_ • Easy integration with conventional wearable/medical devices like hearing aids and headphones
Figure 5(a)	76	Cognitive state monitoring, blood flow measurement	RTD array (4 × 4) (thin film Au), soft flexible sensors with polyimide and silicone encapsulation	Epidermal	Single-ended Multi-point continuous time	• Sensor array enables spatiotemporal mapping of perfusion in capillaries and blood flow through arteries • Skin temperature can be correlated with different cognitive states
Figure 5(b)	117	Cognitive state monitoring	IR camera imaging	Non-contact/facial map	Single-ended Multi-point continuous time	• Temperature mapping of facial skin temperature can allow for identification of different cognitive states

Ota *et al.* developed a wireless, 3D-printed device mounted on the ear for the measurement of *T*_core_ [Fig. 4(b)]. The device consists of a thermistor, which measures environmental temperature (*T*_E_) and an infrared detector which measures the differential temperature between *T*_E_ and the tympanic membrane inside the ear. The tympanum is less susceptible to environmental changes compared to the skin, making it an ideal location for sensing *T*_core._^141^ Tests on a subject during exercise demonstrate excellent agreement between the device developed in this work and a commercial tympanic membrane temperature sensor, while skin-mounted temperature sensors exhibit no such correlation. Experimental demonstrations of varying environmental temperature have significant impact on skin temperature, but no significant changes in tympanic temperature. The authors integrate the device with a bone-conduction hearing aid, illustrating multifunctional use and ease of integration with conventional medical/wearable devices (e.g., hearing aids, headphones).^141^

**FIG. 5.**
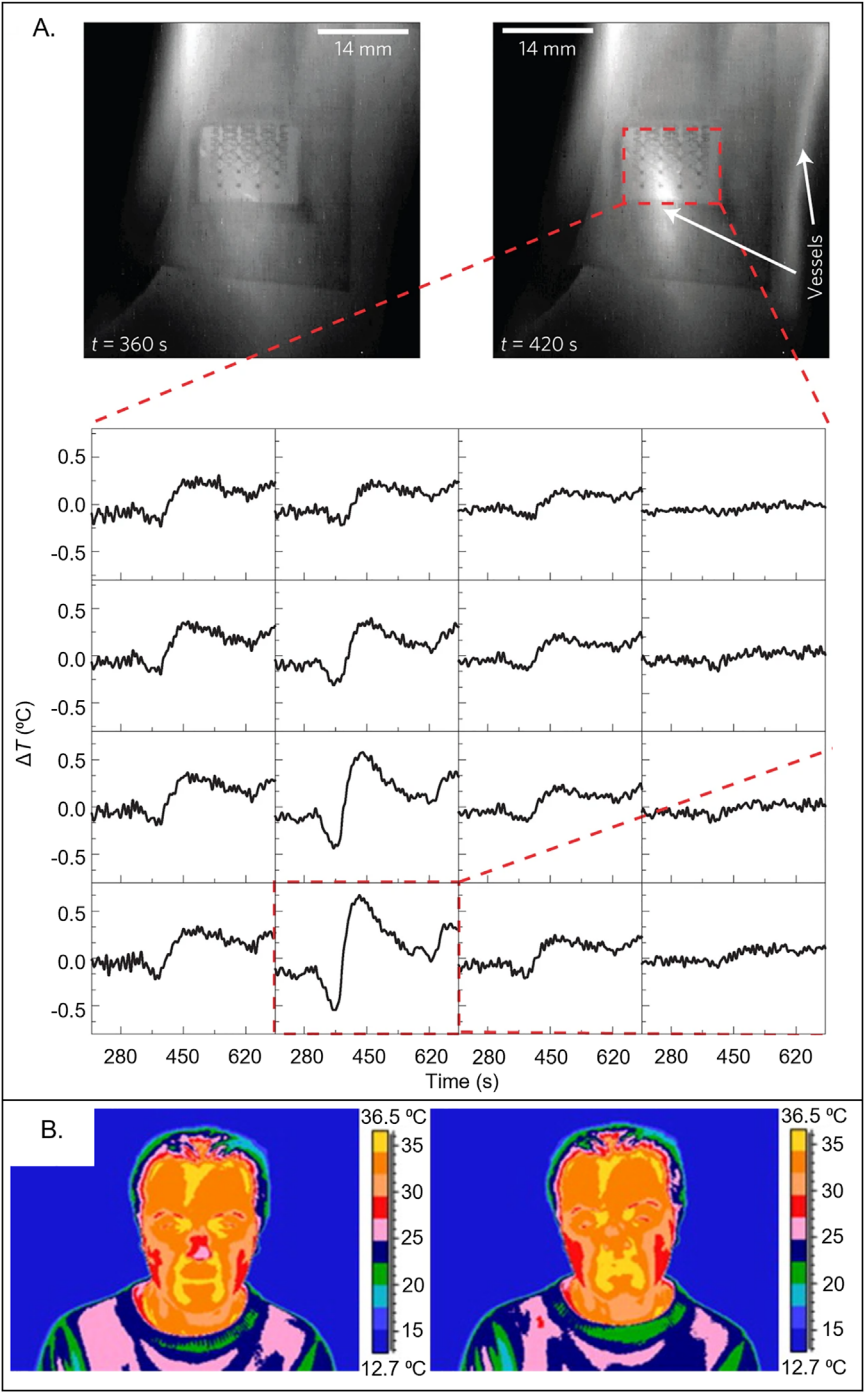
Temperature mapping of the skin surface. (a) Infrared camera images (top) and corresponding temperature measurements from an array of soft, epidermal temperature sensors during a reactive hyperemia test. (b) Infrared camera images of a subject before (left) and after (right) experiencing high emotional arousal stimuli. (a) Reproduced with permission from Webb *et al.*, Nat. Mater. **12**, 938–944 (2013). Copyright 2013 Springer Nature. (b) Reproduced with permission from Salazar-Lopez *et al.*, Conscious. Cognit. **34**, 149–162 (2015). Copyright 2015 Elsevier.

Spatiotemporal mapping of the temperature of the skin surface can lend insight into a subject's health status and cognitive state. Webb *et al.* introduced arrays of epidermal temperature sensors to map the surface of the skin, with examples in measuring blood flow and/or detecting subtle spatiotemporal fluctuations **[**Fig. 5(a)]. Low frequency oscillations (0.005–0.5 Hz) of magnitudes of 0.1–1.0 °C detected by these sensors on the skin of the palm follow from changes in blood perfusion due to the sympathetic activity of arteriovenous anastomoses, relevant for conditions, such as congestive heart disease and tissue hypoxia.^76^ A reactive hyperemia test with a sensor array placed above the ulnar artery demonstrates the use of temperature as a metric for changes in blood flow [Fig. 5(a)]. The experiment involves a blood pressure cuff placed on the upper arm to temporarily occlude blood flow. During occlusion, the temperature decreases due to the absence of blood flow ( ≈ –0.4 °C). After the release, the temperature exceeds the baseline value ( ≈ +0.8 °C) before returning to normal. Sensors placed near the artery exhibit the greatest change while locations away from the artery show no significant change (∼0–0.2 °C), illustrating the importance of temperature mapping to detect spatiotemporal variations.

Cognitive state can contribute to changes in local skin temperature. Epidermal temperature sensors placed on the skin of the palm can detect changes from mental stimuli (mental math), in good agreement with conventional, high-precision infrared cameras.^76^ Another study highlights changes in facial temperature using infrared imaging (n = 40) while presenting subjects with various positive, neutral, and negative visual stimuli [Fig. 5(b)].^117^ A significant change in temperature (−0.85 °C) appears on the tip of the nose compared to the neutral condition (*p* < 0.001) for images with negative, low emotional impact, while a significant increase in temperature (+ 0.96 °C) occurs for images with negative, high emotional impact (*p* < 0.003). Images with strong, positive emotional arousal leads to a + 1.66 °C change in nasal temperature (*p* < 0.001). Similar procedures can assess other emotions, such as empathy and love, of interest in applications in mental health.^117^

## IV. TECHNIQUES FOR MEAURING THE ISOTROPIC, EFFECTIVE THERMAL TRANSPORT PROPERTIES OF THE SKIN

Techniques to measure the isotropic, effective thermal transport properties of the skin involve some combination of thermal power delivery to the skin and sensing the spatiotemporal patterns of temperature that result. Heating methods, like sensing techniques, typically exploit electrical (resistive, or Joule) or optical (absorption) means in a constant mode over the course of the measurement (direct current, DC) or in a time variant (e.g., alternating current, AC) fashion. During Joule heating, a current passes through a resistive heating element in physical contact with the surface a sample of interest for a well-defined time, often on the order of ∼1 s to a few 100 s.^31,73,76,78,79^ Techniques like the 3-omega method involve AC sinusoidal Joule heating, typically with frequencies between 1 and 1000 Hz.^74^ The measurement time (DC) or frequency (AC) and the heater geometry can be used to tailor the effective sensing depth.^78^

A combination of heating and sensing components like those described in previous paragraphs form basis for thermal transport measurements. Heating induces spatial and temporal changes in the distribution of temperature across the sample surface and into its depth. Sensors, typically interfaced to the surface at one or more spatial locations, measure these changes to allow, with suitable models of heat flow, determination of the intrinsic thermal properties. In many thermal transport measurement techniques, a single element serves as both the heater and the sensor. Self-heating induces a change in temperature, the magnitude of which depends on the thermal properties of the surrounding materials. This change in temperature leads to a change in resistance due to the temperature coefficient of resistance of the constituent material. Transient plane source (TPS) measurements in their simplest embodiment exploit a single RTD component that serves simultaneously as a heater and sensor. A recent work demonstrates the use of the TPS technique for evaluations of the skin to a range of characteristics depths (∼50 *μ*m–6 mm), defined by the geometry and the timescales of the measurement, typically with DC power for Joule heating (∼10–60 mW for heater *r* = 0.5–4.5 mm that lead to maximum increases in temperature, ∼1–5 K) that fall far below the thresholds for damage.^75,78^ Another version of this approach relies on one or more separate sensors.^79^ The pulsed decay technique is analogous to TPS but instead observes the temperature decay of the heater after a short (∼3 s) DC heating pulse,^88–143^ most often used to characterize skin at shallow depths (∼0.9 mm).^88^

The 3-omega technique uses a resistive heater with a linear geometry for simultaneous AC heating and sensing. In comparison to DC approaches, measurements made using the 3*ω* technique are less sensitive to radiative and convective heat loss mechanisms (e.g., air convection) and are less affected by boundary conditions between the sample under consideration and its substrate.^74^ The measurement depth is continuously tunable based on adjustment of the heating frequency (< 100 *μ*m for high frequency heating to ∼2 mm for low frequency heating, depending on heater geometry) and enables relatively short measurement times (< few s to tens of s) compared to DC approaches (several seconds—hours); hence, measurements can be collected even with drifts in the baseline temperature associated with the sample or environment.^74,120,144,145^ There are far less constraints for sample size using the 3*ω* technique as compared to TPS owed to the ability to highly confine the measurement depth—the limiting factor is the minimum feature size of the line heater that can be patterned using standard lithographic techniques.^145^ In contrast, DC techniques like TPS typically involve measurement times chosen such that the sample can be treated as a “semi-infinite solid,” where the sample size is sufficiently larger than the thermal penetration depth in each dimension.^146^ Table V summarizes the differences between TPS and 3ω.

**TABLE V.** Comparison between transient plane source and 3-omega techniques.

	Transient plane source	3ω
References	73, 76, and 78	74, 144, and 158
Heating type	DC	AC
Heating frequency *f* (Hz)	⋯	0.1–15000
Heater geometry	Disk/square (2D) *R* = 0.5–4.5 mm	Line (1D) *b* = 200 nm–80 *μ*m *L* = 20 *μ*m–2.5 mm
Heating power density *q*	1–10 W mm^−2^	1.34 W m^−1^
Measurement depth *h* (mm)	0.050–6	<0.005–2
Measurement time (s)	2–300 (depending on *h*)	6.6 × 10^−5^–10 (depending on *ω*)

Photothermal techniques can replace the resistive heating/sensing approaches outlined above for remote, non-contacting measurements. In many examples of this approach, heating of the skin occurs with a laser. Sensing occurs through optical imaging of the skin temperature or by detecting changes in characteristics of the light scattered from the surface of the tissue due to temperature modulation of the refractive index and/or local thermal expansion.^147–149^ Examples of photothermal beam deflection (PBD) methods applied to samples of biological tissue use a *λ* = 632.8 nm “probe” laser to detect changes in the angle of scattered light using a phase sensitive photodetector following local heating from a *λ* = 8–10 *μ*m “pump” laser.^147,148^ Reports show that PBD can probe superficial layers of the skin (∼17–44 *μ*m) with high temporal (few ms to a few 100 ms) and spatial (10–300 *μ*m) resolution.^147,148^ Complex optical setups, dependence on skin tone/pigmentation and sensitivity to motion artifacts represent key disadvantages.^147^ Of these various schemes, electrical methods are generally most practical for continuous measurements of conscious subjects, particularly for use outside of clinical or laboratory settings.^74,79,88,149^ The remainder of this section, therefore, focuses on electrical measurement techniques—TPS and 3-omega—in detail.

In all cases, safe thresholds of heat must be maintained to prevent thermal injury of the subject. The United States Food and Drug Administration (FDA) recommends medical equipment/devices conform to the International Electrotechnical Commission (IEC) standards for maximum allowable temperatures and time for contact of medical electrical equipment with skin. The IEC 6061–1 guideline states that for medical equipment composed of molded material, plastic, or rubber (of relevance in wearable thermal devices) in contact with small areas (< 10%) of the total body area, for applied time *t* < 1 minute, the maximum allowable applied temperature *T*_max_ is 60 °C, for 1 min ≤ *t* < 10 min, *T*_max_ = 48 °C, and *t* ≥ 10 min, *T*_max_ = 43 °C. For applied parts intended to supply heat to a patient, if the applied *T* > 41 °C, additional justification is required.

### A. Transient plane source method

**FIG. 6.**
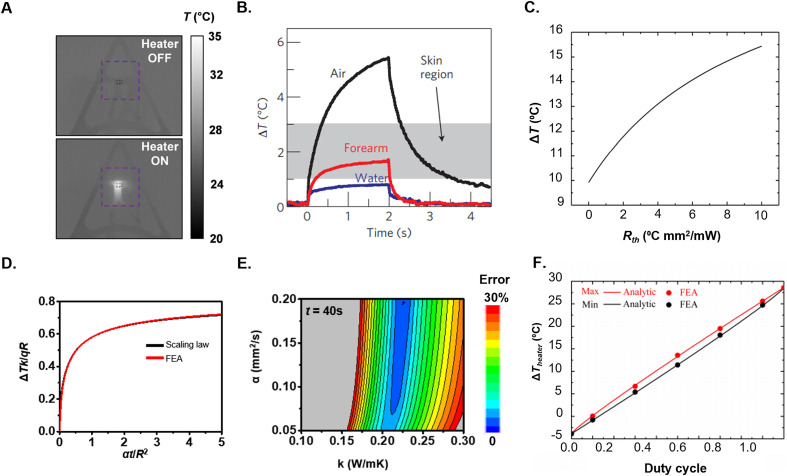
Transient plane source measurements. (a) IR Camera images of a surface mount (SMD) transient plane source (TPS) heater before and after joule heating. (b) Measured ΔT as a function of time for various materials. (c) ΔT as a function of thermal contact resistance R_th_ between the heater and skin. (d) Scaling law relating the measured ΔT to k through known the known values of heater radius R, measurement time t, thermal power density q, and a standard estimate for α (0.15 mm^2^/s). (e) Error contour plots of α vs k for measurement t = 40 s. **(F)** ΔT_heater_ as a function of duty cycle for pulsed heating. (a) Reproduced with permission from Madhvapathy *et al.*, Sci. Adv. **6**, eabd7146 (2020). Copyright 2020 Authors, licensed under a Creative Commons Attribution (CC BY) license. (b) Reproduced with permission from Webb *et al.*, Nat. Mater. **12**, 938–944 (2013). Copyright 2013 Springer Nature. (c) Reproduced with permission from Li *et al.*, AIP Adv. **8**, 055102 (2018). Copyright 2018 Authors, licensed under a Creative Commons Attribution (CC BY) license. (d) and (e) Reproduced with permission from Crawford *et al.*, Extreme Mech. Lett. **22**, 27–35 (2018). Copyright 2018 Authors, licensed under a Creative Commons Attribution (CC BY) license. (f) Reproduced with permission from Cui *et al.*, Micromachines **7**, 210, (2016). Copyright 2016 Authors, licensed under a Creative Commons Attribution (CC BY) license.

For most applications to the skin, TPS involves injecting a direct current *I* through a thin film metal (or SMD) heater of resistance *R* and radius *r* for a fixed time *t*, causing Joule heating with power *P* [Fig. 6(a)].

$$P=I^{2}R.$$ (15)

The change in *R* of the heater (Δ*R*) recorded during the heating period relates to the change in temperature through the TCR [Eq. (10)]. Qualitatively, Δ*T* increases rapidly at short times and then approaches a quasi-steady state value Δ*T*_sat_, reflecting the transient temperature response of the material to the applied thermal power, followed by the quasi-steady state behavior of the material after a sufficiently long time, *t* > *t*_sat._^78^ The average temperature change for a spiral-shaped disk of radius *r* is given by^146^

$$\bar{\Delta T\left(\tau \right)}=P{\left(\pi^{\frac{3}{2}}rk\right)}^{-1}D\left(\tau \right),$$ (16)

where $\tau =\sqrt{t\alpha /r^{2}}$, and

$$D\left(\tau \right)=\int_{0}^{\tau }\frac{d\sigma }{\sigma^{2}}\int_{0}^{1}\mathrm{udu}\int_{0}^{1}\mathrm{vdv}\times \exp\left(-\frac{u^{2}+v^{2}}{4\sigma^{2}}\right)I_{0}\left(\frac{uv}{2\sigma^{2}}\right),$$ (17)

where *τ* is a dimensionless measure of time, and *I*_0_ is the second-order Bessel function of the zeroth kind. The parameters *u* and *v* represent 2D Fourier space. Equations (16) and (17) can be solved using Finite Element Analysis (FEA). Simplified scaling laws [Eqs. (18) and (19)], with analytical solutions solved through FEA relate the Δ*T* and *k:*^73^

$$\frac{\Delta Tk}{qr}=f\left(\tau \right),$$ (18)

where

$$f\left(\tau \right)=\frac{2qR}{k}\int_{0}^{\infty }\left\{{\left[J_{1}\left(x\right)\right]}^{2}\mathrm{erf}\left(x\sqrt{\tau }\right)\right\}\frac{dx}{x^{2}}.$$ (19)

*J*_1_ is the first-order Bessel function of the first kind. Equations (18) and (19) assume a solid disk shape, as compared to Eqs. (16) and (17), which assume a spiral geometry. The difference between these two cases can be neglected in most instances.^73^ Eqs. (18) and (19) demonstrate that Δ*T*, *q, k*, and *r* have linear co-dependence, while *t* and *α* have non-linear relationship to the measured Δ*T*. For applications to skin, *q* is chosen such that Δ*T* < 10 °C (typically < 5 °C), the threshold for sensation of pain and for compliance with IEC6061–1.^76,78,150^

Representative TPS data collected from three materials (water, air, and the skin of the forearm) appear in Fig. 6(b). The results display the expected inverse relationship between Δ*T* and the measurement time *t.*^76^ The gray region of the graph indicates the full range of Δ*T* observable for skin with a 1 mm^2^ square heater at *q* = 1 mW for *t* = 0–2 s.^76^ Figure 6(c) summarizes modeling results for the change in the temperature of the heater Δ*T*_heater_ with respect to thermal contact resistance *R*_t_ with the skin. Δ*T*_heater_ increases monotonically with respect to *R_t_*, as expected, as air gaps fill voids between the skin and the heater.^123^ The presence of air, due to its low thermal conductivity (*k*_air_ = 0.02 W/m K) leads to an increase in Δ*T*_heater_. The Δ*T* can be used to compute *k* of the skin using FEA or the simple scaling law presented in Eqs. (18) and (19), as graphically represented in Fig. 6(d) for known *q*, *R*, and *t.*^73^ Here, the extraction of *k* assumes the skin across the measurement depth can be considered as an isotropic, homogenous medium. This scaling law also assumes an estimated *α*_skin_ = 0.15 mm^2^ s^−1^ ± 20%.^73^ For large values of *t* and small values of *R*, the value of *α* has minimal impact on Δ*T*, and $\frac{\Delta Tk}{qR}$ is nearly constant. Figure 6(e) illustrates that the error in fitted values of *k* due to uncertainties in *α* is minimized in this quasi-steady state regime.^73^ The blue region of the contour plot (where FEA fitting error is minimum) is vertical, indicating that an accurate value of *k* (in this case, ∼0.21 W/m K) can be extracted independently of the value of *α* (0.05–0.2 mm^2^ s^−1^) at *t* = 40 s.

Drift of baseline temperature *T*_0_ of the skin represents an additional source of error for measurements of Δ*T.*^151^ In the case of inflammatory diseases, such as urticaria, lesions can develop over the course of minutes. Changes in the temperature of the environmental temperature can vary over similar timescales. Pulsing the heater allows for re-calibration of *T*_0_ for every pulse, but this approach effectively reduces the duty cycle and the effective measurement depth [Fig. 6(f)].^152^ Measurements of the temperature of adjacent, unheated, regions of the skin can, in certain cases, be used to eliminate these drifts via a differential mode of detection.

Understanding the effective measurement depth, *h*, is important because the skin is a layered structure with depth-dependent properties, as discussed in Sec. II. For the TPS technique, this measurement depth is comparable to the thermal penetration depth, *h* ∼ $\sqrt{\alpha t}$, where *α*_skin_ ≈ 0.15 mm^2^ s^−1^.^75,146^ Examples of the TPS technique applied to skin involve depths between ∼100 *μ*m and ∼6 mm, dictated by the sizes of the heaters and the timescales for the measurements.^75,78^ This range spans the stratum corneum, the epidermis, dermis, and hypodermis.

**FIG. 7.**
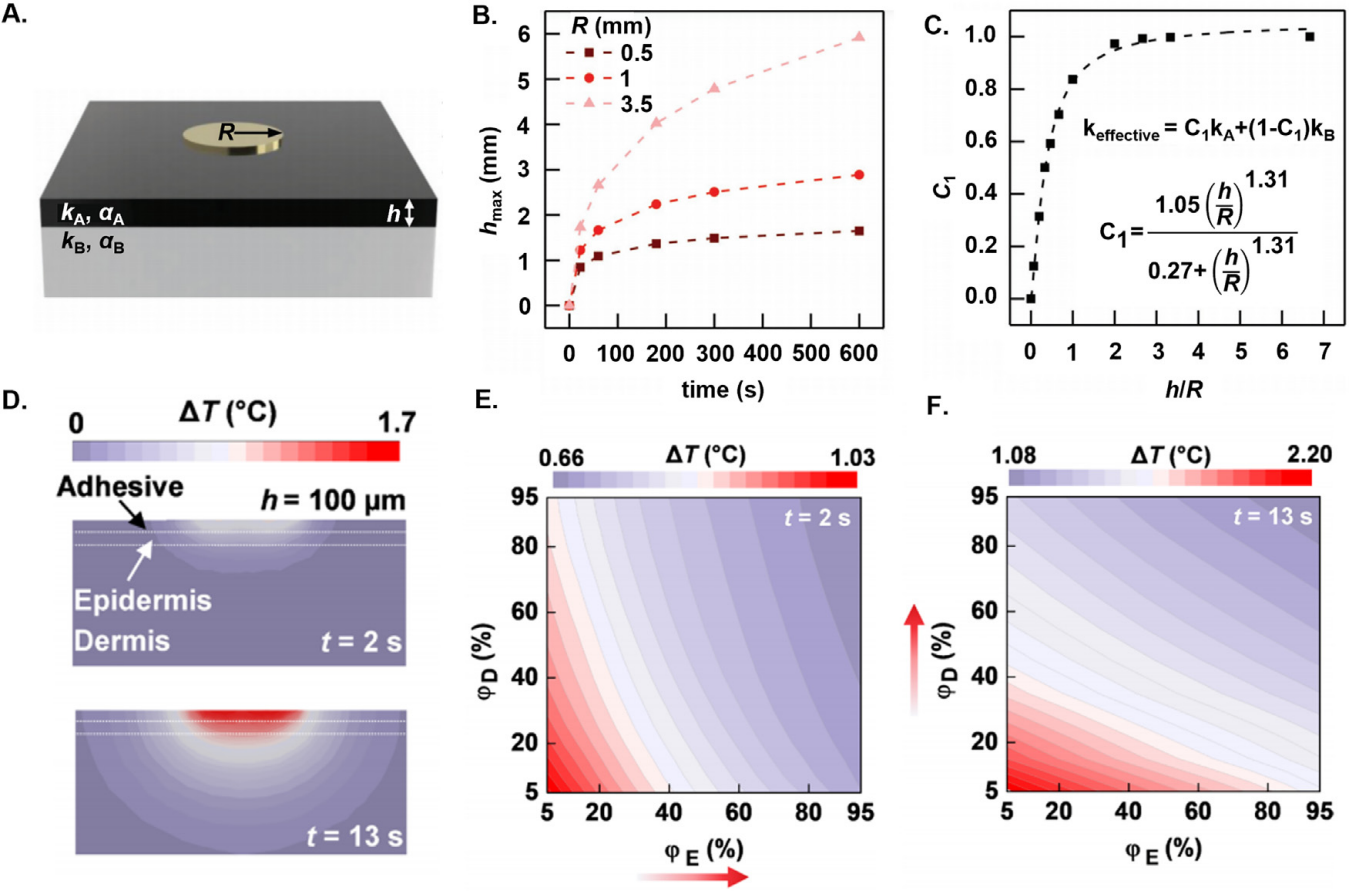
Measurement of the thermal properties of the individual layers of skin using transient plane source. (a) Schematic image of a TPS heater of radius R on top of a bilayer model of skin approximating the epidermis (black) and dermis (gray) with thermal properties k_A_, α_A_, and k_B_, α_B_, respectively. h is the thickness of the top layer of the skin. (b) Relationship between the maximum depth sensitivity h_max_ and t for various R. (c) Effective thermal conductivity model illustrating skin thermal properties as a weighted average of its constituent layers. C_1_ is a constant dependent upon on the ratio of the maximum desired measurement depth h to the heater radius. (d) Heat profiles into the skin for an epidermal thickness of 100 *μ*m for a measurement time of 2 and 13 s. (e) Contour plot of the measured ΔT illustrating the water content of the dermis as a function of the epidermis for t = 2 and (f) t = 13s. (a)–(c) Reproduced with permission from Madhvapathy *et al.*, Adv. Funct. Mater. **28**, 1802083 (2018). Copyright 2018 Wiley-VcH. (d)–(f) Reproduced with permission from Madhvapathy *et al.*, Sci. Adv. **6**, eabd7146 (2020). Copyright 2020 Authors, licensed under a Creative Commons Attribution (CC BY) license.

Models that account for the skin as bi-layer systems allow the extraction of the approximate thermal properties of the individual layers. Recent research (2018) treats the skin as a bi-layer made up of the epidermis/dermis (depicted as layer A, with thickness *h*) and hypodermis (depicted as layer B) [Fig. 7(a)] in series of TPS measurements with different heater sizes and measurement times. Increasing the size and the time increase the measurement depth [Fig. 7(b)]. For a single-layer model, the *k* can be denoted as “*k*_eff,_” as a weighted, volumetric average of *k*_A_, the thermal conductivity of the epidermis/dermis, and that of the hypodermis, *k*_B_. Figure 7(c) illustrates a constant C_1_ that defines the weight fraction of the volume of heat that penetrates through layer A.

Fitting measurements to FEA results can determine the thermal properties of the epidermis and dermis, using devices and measurement parameters to restrict the characteristic depth to ∼1 mm.^79^ One reported approach uses a small (∼1 × 1 mm^2^) heater and an analysis of the dependence of Δ*T* on *t*. At short times (*t* = 2 s), the heat propagates mostly into the epidermis and at longer times (*t* = 13 s), a larger portion of the heat propagates into the dermis [Fig. 7(d)]. A micromechanics model treats the thermal properties as a composite of pure dry tissue and water to determine the volumetric water content *φ* in the skin. Contour plots of Δ*T* for varying *φ*_E_ (epidermal water content) and *φ*_E_ (dermal water content) at different times enable separate the extraction of the water content of the epidermis and dermis, respectively [Figs. 7(e) and 7(f)].

### B. 3-omega technique

The 3-omega technique involves injection of an alternating current (AC) *i* at angular frequency *ω* = 2π*f* into a one-dimensional (1D) heater of length *l*:

$$i(t)=i_{0}\text{sin}(\omega t).$$ (20)

Typically, *f* ranges between 1 and 1000 Hz. This alternating current induces Joule heating:

$$P(t)=i^{2}R=i_{0}^{2}R{\text{sin}}^{2}\left(\omega t\right)=P_{\mathrm{rms}}(1-\text{cos}\left(2\omega t)\right),$$ (21)

where

$$P_{rms}=\frac{1}{2}i_{0}^{2}R.$$ (22)

*P*_rms_ represents the root mean square (RMS) power dissipated over one heating cycle, and *I*_rms_ is the corresponding RMS heating current. The total power can be separated into two Joule heating components, the DC component *P*_rms_ and the AC component $P_{\mathrm{rms}}\text{cos}2\omega t$.

From the literature, the steady-state harmonic temperature oscillations in the metal heater produce harmonic variations in the resistance of the metal line given by the following equation:^153^

$$R\left(t\right)=R_{0}(1+\beta \Delta T_{DC}+\beta \left|\Delta T_{AC}\right|\text{cos}(2\omega +\phi )),$$ (23)

where *β* is the TCR of the metal heater, Δ*T*_DC_ is the temperature change due to the RMS power dissipated by the 1D line heater, |Δ*T*_AC_| is the magnitude of the temperature oscillations due to the AC Joule heating component, and ϕ is the phase delay between the temperature oscillations and the injected current *i*. The voltage across the 1D heater is the product of the heater resistance and the current injected into the heater:^153^

$$V\left(t\right)=i_{0}R_{0}[\left(1+\beta \Delta T_{DC}\right)\text{cos}\left(\omega t\right)+\frac{1}{2}\beta \left|\Delta T_{AC}\right|\text{cos}\left(\omega t+\phi \right)+\frac{1}{2}\beta |\Delta T_{AC}|\text{cos}(3\omega +\phi )].$$ (24)

The 3*ω* component of the voltage arises from the product of the resistance change due to Joule heating at 2*ω* and the input current oscillating at frequency *ω*. The amplitude of the third harmonic voltage is given by^153^

$$|V{}_{3\omega }|=\frac{1}{2}V_{0}\beta |T_{2\omega }|,$$ (25)

where *V*_0_ = *i*_0_*R*_0_ and *T*_2ω_ is the complex AC temperature oscillation at 2ω. The average temperature of the line heater is given by^74^

$$\bar{\Delta T}=\frac{P}{l\pi k}\int_{0}^{\infty }\frac{{\text{sin}}^{2}vb}{{\left(vb\right)}^{2}{\left(v^{2}+q^{2}\right)}^{1/2}}dv=2\beta \frac{{|V}_{3\omega }|}{i_{0}},$$ (26)

where *l* and *b* are the length and half-width of the line heater, respectively, *v* represents 1D Fourier space, and 1/*q* =* h* which is the thermal penetration depth. *h* is given by^74^

$$h={\left(\frac{\alpha }{i2\omega }\right)}^{\frac{1}{2}}.$$ (27)

For typical values of *ω* (2π*f* where *f* = 1–1000 Hz), line heaters of metal width = 5 *μ*m, thickness = 100 *μ*m, and *L* = 1.5 mm, and for *α*_skin_ = 0.15 mm^2^ s^−1^, *h* does not exceed 100 *μ*m. The 3-omega technique is ideal for sensing across shallow depths of the epidermis, with potential applications in skin hydration. At very low frequencies (e.g., 0.1 Hz), *h* = 1 mm can be achieved. In such cases, however, boundary conditions become significant, and the substrate (*t*_substrate_) and sample thicknesses (*t*_sample_) must satisfy a semi-infinite condition: *t*_substrate_/*h* > 5, *t*_sample_/*h* > 5 for < 1% error in measured *k* of the sample.^144^

**FIG. 8.**
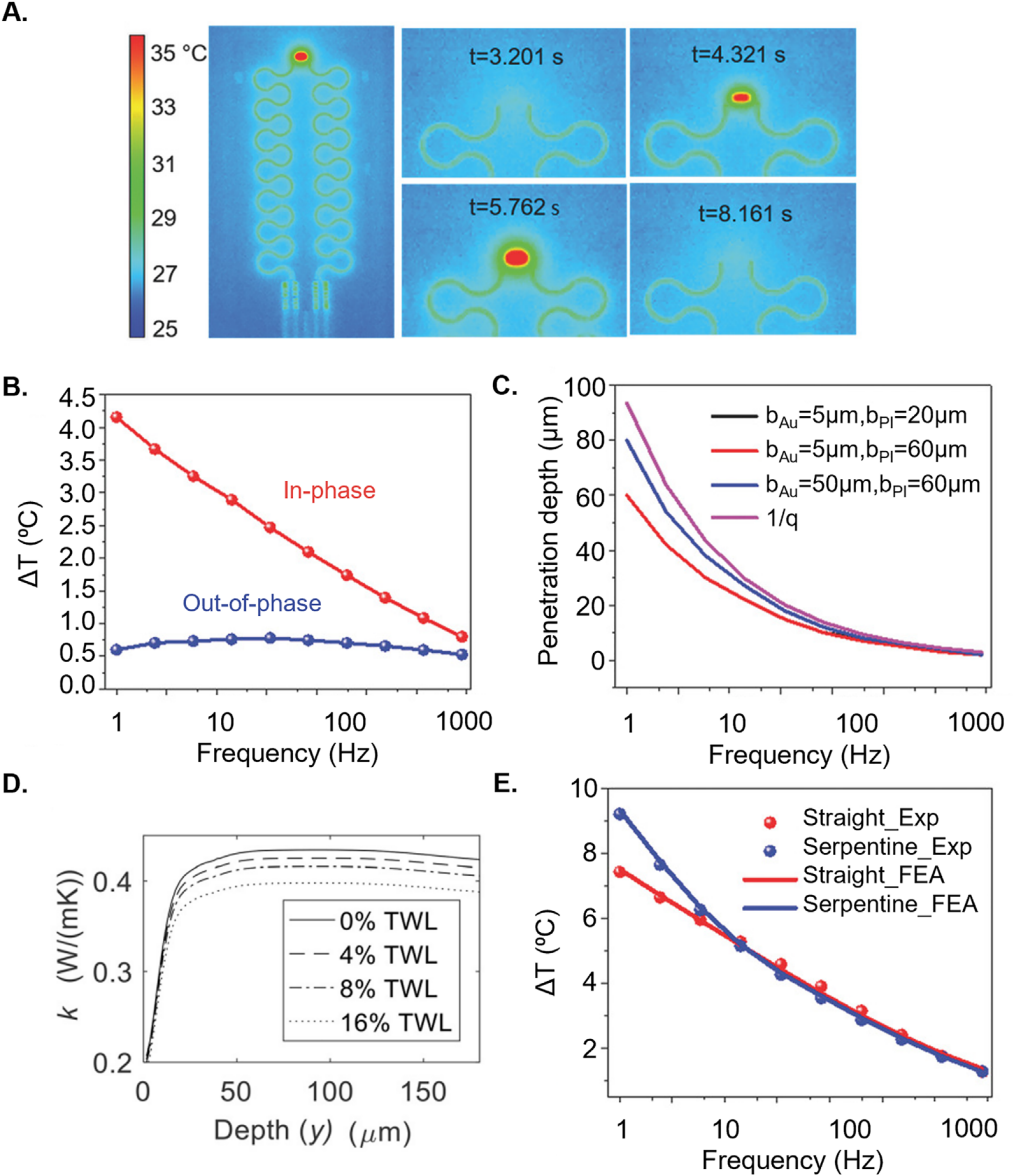
3-omega measurements. (a) Infrared camera images of the 3-omega heater at various time points during AC heating. (b) Experimental relationship between the magnitude of the real (in-phase) and imaginary (out-of-phase) components of ΔT as a function of heating frequency. (c) Relationship of the penetration depth of the thermal wave into the skin as a function of frequency for various geometrical considerations. (d) FEA simulations of k as a function of penetration depth for various hydration states of the skin. (e) FEA simulations and experimental measurements of the in-phase ΔT as a function of heating frequency for straight-line and serpentine heater geometries. (a)–(c), and (e) Reproduced with permission from Tian *et al.*, Adv. Funct. Mater. **27**(26), 1701282 (2017). Copyright 2017 Wiley-VcH and (d) Reproduced with permission from Sun *et al.*, J. Heat Transfer 141, 1 (2018). Copyright 2018 ASME.

Tian *et al.* demonstrated soft, stretchable 3-omega sensors for use on the skin.^74^ Fig. 8(a) illustrates the AC temperature oscillations of the Joule heating at 2*ω* frequency. The magnitude of Δ*T* changes with the physiological properties of the skin, such as water content. The out-of-phase (imaginary) component of Δ*T* (*V*_3ω, ip_) directly relates to *k*^144^

$$k_{\mathrm{total}}=\frac{\beta R^{2}i^{3}}{8LV_{3\omega ,op}},$$ (28)

where

$$k_{\mathrm{total}}=k_{\mathrm{sample}}+k_{\mathrm{substrate}}.$$ (29)

The in-phase component (*V*_3ω, ip_) of the measured Δ*T* represents, however, a more reliable measure of *k* [Fig. 8(b)]:^144,154^

$$k_{\mathrm{total}}=\frac{\beta R^{2}i^{3}}{4\pi L}\left(\frac{d\ln\left(\omega \right)}{dV_{3\omega ,ip}}\right).$$ (30)

Equation (30) illustrates that *k*_total_ is directly proportional to the slope of the measured ln(*ω*) vs *V*_3_*_ω_,_ip_* curve—all other parameters are constant/pre-determined within the measurement setup. The thermal diffusion length for the line heater is small for various relevant *ω*; thus, the thermal penetration depth does not surpass ∼100 *μ*m for most line heater geometries investigated in the work of Ref. 74 [Fig. 8(c)]. The 3-omega technique can, however, allow depth profiling of the epidermis. Theoretical simulations reveal that epidermis at various water concentrations can be visualized as a function of measurement depth at different excitation frequencies [Fig. 8(d)].^155^ The backside boundary condition can be exploited to measure tissue thickness below a characteristic excitation frequency, where *h* is no longer confined within the sample.^144^ 3ω sensors can also be used to sense tissue contact, where *V_3_*_ω_ has a value 22% lower than air when in contact with skin, which is 20 × higher than the difference sensed using *V*_1ω._^74^

A key point of interest in Ref. 74 is the use of a serpentine geometry for the line heater (width of Au = 5 *μ*m, width of PI = 20 *μ*m) for stretchability (54% and 71% latitudinal and latitudinal elastic strain, respectively).^74^ These heaters, unlike those with straight-line geometries, are subject to proximity effects typically at low frequencies (<10 Hz), where local thermal oscillations in one part of the heater can impact neighboring sections [Fig. 8(e)].^74^ The effect can be illustrated by two parallel line heaters at a separation distance *D*, where thermal oscillations measured across one line heater are a superposition of its own oscillations and parallel line heater:^74^

$$\Delta T\left(x,t\right)=A\frac{1-\text{cos}\left(2\omega t-\frac{\pi }{4}\right)}{2}+B\frac{1-\text{cos}\left(2\omega t-\frac{\pi }{4}-x\sqrt{\frac{\omega }{2\alpha }}\right)}{2},$$ (31)

where *A* is the magnitude of the thermal oscillation determinable from Eq. (26) and B depends on the oscillation strength as a function of distance from the heater, which can be computed using numerical analysis. Another reported study describes 3ω measurements of *k* using 2D fractal heaters (Peano curve motif), likely also exhibiting proximity effects.^120^

### C. Applications of thermal sensing techniques for studying the isotropic, effective thermal properties of the skin

**FIG. 9.**
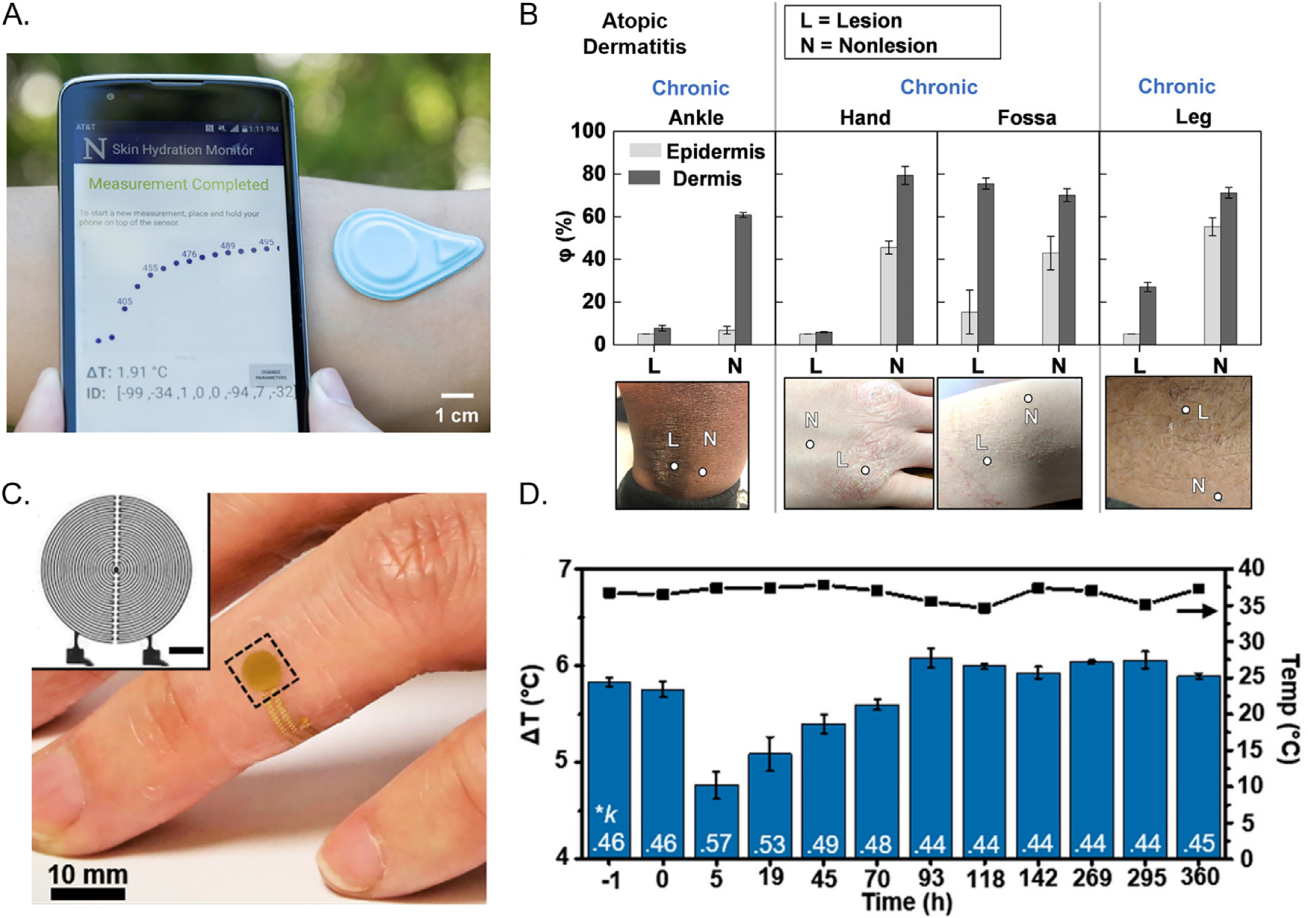
Transient plane source measurements on skin lesions. (a) Image of a wireless, battery-free skin hydration sensor that employs the TPS technique and corresponding cell phone readout, accompanied by (b) measurements of the epidermal and dermal hydration level of the epidermis and dermis on four atopic dermatitis lesions. L indicates the area of the atomic dermatitis lesion and N indicates the non-lesional area with normal appearance adjacent to the diseased location. (c) Photograph of an epidermal TPS sensing platform mounted on a subject's finger. (d) Skin surface temperature and measured ΔT for different time points after an induced sunburn using the sensing platform in (c). Reproduced with permission from (a) and (b) Madhvapathy *et al.*, Sci. Adv. **6**, eabd7146 (2020). Copyright 2020 Authors, licensed under a Creative Commons Attribution (CC BY) license. (c) and (d) Reproduced with permission from Crawford *et al.*, Extreme Mech. Lett. **22**, 27–35 (2018). Copyright 2018 Authors, licensed under a Creative Commons Attribution (CC BY) license.

Measurements of isotropic heat transport based on the methods discussed in Secs. IV A and IV B are of increasing interest for uses in wearable and non-wearable devices. Table VI highlights several representative applications. A recent study (2020) introduces wireless, battery-free sensors that infer hydration (*φ*) based on thermal conductivity measured through interfaces to the skin that exploit near field communication (NFC) links to smart phones [Fig. 9(a)]. These sensors are thin (∼2.1 mm), flexible, and compact (3.1 × 4.5 cm^2^) with the ability to gently mount across multiple body locations. The measurement determines the water content of the epidermis and dermis using the TPS technique. A micromechanics model which treats the skin as a composite of water in a tissue matrix relates the effective thermal properties of the skin to water content:^79^

$$\frac{k}{k_{\mathrm{dry}}}=\frac{\left(p+2\right)+2(p-1)\varphi }{\left(p+2\right)-(p-1)\varphi },$$ (32)

where

$$p=\frac{k_{w}}{k_{\mathrm{dry}}}.$$ (33)

Here, *k*_dry_ and *k*_w_ are the thermal conductivities of dry skin and water, respectively. The sensing electronics and NFC receiving coils define the overall size of the device. The sensor dimensions (comprised of two SMD resistors, ∼0.9 × 0.9 mm^2^ and one NTC thermistor 0.6 × 0.3 mm^2^) are limited by the SMD components.^79^ Thin, patterned metal traces can circumvent these limitations and improve measurement sensitivity.^76^ The results enable assessments of patients with various inflammatory diseases, including atopic dermatitis, psoriasis, urticaria, rocasea, and xerosis cutis. Figure 9(b) showcases measurements captured from patients with atopic dermatitis, a disease of the epidermis. The data reveal differences (average of ∼20%) in epidermal hydration levels of diseased sites (denoted by “L”) in comparison to sites of normal skin (denoted by “N”).^79^ Some patients also display differences in dermal hydration level, indicating possible epidermal thickening. These and other insights can be important in guiding protocols for care of these diseases.

**TABLE VI.** Representative applications of thermal sensing techniques for studying the isotropic, effective thermal properties of the skin.

Figures (Reference)	Application	Sensor geometry	Sensing location	Sensing Technique	Measurement time/freq	Heating power	Measurement depth	Key details
Figures 9(a) and 9(b) (Ref. 79)	Skin hydration, skin disease diagnosis	SMD resistor heaters (2 × 0.81 mm^2^) and thermistor (1 × 0.18 mm^2^) sensor	Epidermal	TPS (*k*_skin_, *T*_skin_)	13 s	10 mW/mm^2^	∼ 1 mm	• Thin, wireless (NFC), battery free wearable device • Micromechanics model enables the extraction of epidermal and dermal hydration independently • Quantitative diagnosis of the skin diseases like atopic dermatitis, psoriasis, rosacea, urticaria, and xerosis cutis
Figures 9(c) and 9(d) (Ref. 73**)**	Erythema recovery	Thin film Au RTD with polyimide and silicone encapsulation (r_heater_ = 2 mm)	Epidermal	TPS (*α*_skin_, *k*_skin_, *T*_skin_)	2–60 s	2–3 mW/mm^2^	…	• Elevated skin perfusion following sun exposure, and subsequent healing can be quantitatively tracked by monitoring *k*_skin_ and *T*_skin_ • TPS measurements are also able to identify blood flow changes resulting from local heating or cooling of the skin
^78^	Study of the skin thermal properties as a function of skin depth	Dual thin film Au RTDs with polyimide and silicone encapsulation (r_heater_ = 0.75 mm, 3.5 mm)	Epidermal	TPS (*α*_skin_, *k*_skin_, and *T*_skin_)	60 s	1–3 mW/mm^2^	Up to 6 mm	• Soft skin conformable stretchable sensors with varying heater-sensor radii and variable measurement time enable the measurement of skin thermal properties up to a depth of 6 mm
Figures 10(a) and 10(b) (Ref. 120)	Cutaneous wound healing	Thin film Cu RTD linear array with polyimide/silicone encapsulation (array of 6, 4.5 × 4.5 mm^2^ heater/sensors)	Epidermal	3*ω* (*k*_skin_, *T*_skin_)	1–3 Hz	∼ 0.5 mW/mm^2^	0.1–1 mm (dependent on freq)	• *k* for epidermis and dermis correlates directly with blood flow, enzymatic reactions, and tissue healing
^157^	Wound healing, diabetic foot ulcers	FLIR ONE thermal camera attached to smartphone	Non-contact	IR imaging (*k*_skin_, *T*_skin_)		N/A	Superficial (∼ epidermis only)	• *k*_skin_ is analytically extracted from IR images. Costly and requires patient immobilization • Skin pigmentation may impact measured temperature accuracy due to varied absorption/reflection of IR light
Figures 11(a) and 11(b) (Ref. 88)	Characterization of malignant melanoma lesions	Guard-heater thermistor probe in pen-like format	Epidermal	Pulse-power delay (similar to TPS) (*k*_skin_, *T*_skin_)	3 s pulsed heating + 3 s cooling	∼ 20 mW/mm^2^	Up to 10 mm (maximum thickness of studied tumor)	• *k*_effective_ for melanoma lesions > non-lesional areas • Linear relationship found between tumor thickness and *k*_skin_ • Fast, simple, and noninvasive characterization relative to skin biopsy and imaging techniques
Figures 11(c) and 11(d) (Ref. 159**)**	Characterizing *k* of single cancerous cells	Nanoscale heater-sensor (half-width ∼ 200 nm)	Non-body, single cell sample analysis	3*ω* (*k*_cell_)	500–15 kHz		∼5–10 *μ*m	• Nanoscale heater patterned using e-beam lithography allows for the determination of small volumes (∼ 10 pl) and small effective depths (∼10 *μ*m) like single cancerous cells Normal cells have ∼ 2.2% higher k than basal cell carcinoma cells
^74^	Skin perfusion analysis	Thin film Au heater (width ∼ 5 *μ*m, 100 nm thick) with PI/silicone encapsulation	Epidermal	3*ω* (*α*_skin_, *k*_skin_)	1–1000 Hz	∼ 268 mW/mm^2^	Up to 80 *μ*m (dependent on freq)	• (*α*_skin_, *k*_skin_) are very close proxy for skin perfusion. Hyperemia or reddening of the skin is monitored as a function of time
Figures 12(a) and 12(b) (Ref. 160)	Heat therapy and impact on skin thermal properties	Thin film Au heater (∼200 nm thick) with PI/silicone encapsulation	Epidermal	TPS (*k*_skin_, *T*_skin_)	20 min	…	…	• Measurement of skin properties is used in a closed loop system to control the heating of underlying skin and thereby the transdermal diffusion rate of drugs for therapeutic purposes

Alternative versions inspired by Ref. 79 replace NFC powering/communication with Bluetooth Low Energy (BLE) for wireless data transfer, powered by a lithium polymer battery.^156^ A modified sensing structure uses NTC thermistors placed above the heater, thermally coupled together through a 75-*μ*m-thick layer of PI, the insulating “body” of the flexible printed circuit board. The heater was embedded in a 180-*μ*m adhesive silicone gel layer, compared to afore-mentioned structures where the heater is exposed to air on one side. Because this sensing platform contains the same materials and corresponding thermal masses (PI, copper traces, silicone adhesive), the top/bottom heater/thermistor structure offers no improvement in sensitivity to the lateral structure (i.e., for a given *q*, both structures exhibit the same range of Δ*T* for *φ* = 0% –100%). Additional thermistors used in the system to decouple the effects of changes in skin and environmental temperatures on Δ*T* do not assist in improving the system response time, which is dependent upon the thermal masses of the heating/sensing components and encapsulation layers.

Crawford *et al.* demonstrate soft, epidermal TPS sensors can be used for quantitative assessment of erythema recovery time from (1) solar radiation, causing sunburn, (2) heating of the skin with a source of thermal power, and (3) cooling of the skin with an ice pack [Fig. 9(c)].^73^ The authors used two metrics, *k* and skin surface temperature *T*, to quantify erythema with a TPS sensor with *r*_heater_ = 2 mm and measurement *t* = 60s for *q* = 2 mW/mm^2^. In case (1), prior to exposure to direct sunlight (UV index = 9) for one hour, the values of *k* and *T* for the skin were 0.46 W/m K and 36.5 °C, respectively, with only minor changes immediately after exposure [Fig. 9(d)]. When visual redness was greatest (*t* = 5 h after exposure), *k* = 0.57 W/m K and *T* ≈ 37.5 °C, due to elevated levels of near-surface blood flow. The values returned to baseline ∼4 days after exposure. The measured *k* of the skin showed similar correlations with the visual appearance of the skin for heat-induced erythema in case (2) but not in case (3). Reference 78 reports a similar experiment to case (3) using the application of a cold pack to the skin for ∼10 min to four subjects, resulting in lower measured *k* after the cold pack is removed using two sensors at different measurement depths (1.42 and 2.66 mm). The difference in agreement between the experiments in Refs. 73 and 78 may be due to subject-to-subject variations. This work provides a foundation for further investigations of thermal sensing as a quantitative basis for evaluating erythema recovery on a large population scale.

**FIG. 10.**
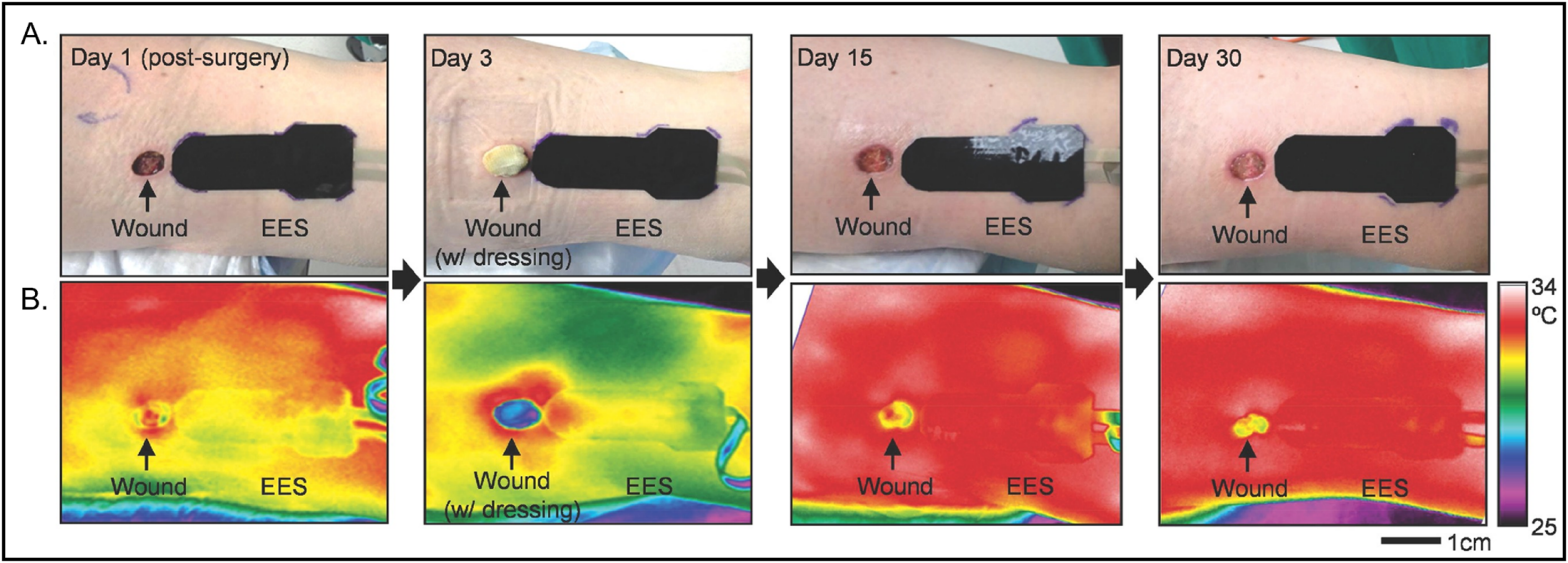
Epidermal electronics system using the 3ω technique for cutaneous wound healing. (a) Photographs and (b) infrared camera images of a thermal EES system placed next to a granulating wound over 30 days post-surgery. The EES consists of six epidermal 3ω heaters spaced equidistantly in a linear geometry for multi-point mapping of skin surface temperature and thermal conductivity adjacent to a wound site. Variations in the visual appearance of the wound, along with skin temperature and thermal conductivity, can be used to characterize inflammation during the healing process. Reproduced with permission from Leleux *et al.*, Adv. Healthcare Mater. **3**, 1377–1380 (2014). Copyright 2014 Wiley-VcH.

Cutaneous wound healing represents an additional application where thermal transport sensing offers quantitative information compared traditional practices for the assessment and care of wounds (e.g., clinical visual inspection). Hattori *et al.* developed multimodal thermal sensors (measurements of both skin surface temperature and *k*) that laminate gently and reversibly to the perilesional site of a cutaneous wound without causing irritation compared to conventional medical tape [Fig. 10(a)]. The 3ω technique at two different angular frequencies (1 and 3 Hz) allows for sensitive measurements of *k* for both the epidermis and dermis, important in detecting changes in blood flow during tissue healing. Studies conducted on patients with incisional cutaneous wounds over a 30-day period using an array of six equidistantly spaced sensors (total length = 45 mm) reveal that both skin surface temperature and *k* at different distances from the wound are useful in detecting inflammation during the healing process, resulting from increased blood flow and enzymatic reactions [Fig. 10(b)].^120^ In contrast, the contralateral location on the body, serving as a reference to the wound site, does not exhibit significant variations in temperature. On-going work focuses on device layouts that allow direct lamination over the sensitive and complex geometry of the wound site, along with options for incorporating additional, complementary sensing techniques (e.g., pH, TEWL).^120^

Another sensing approach for quantitative monitoring of wounds utilizes infrared imaging. As noted in Sec. III B, imaging techniques are attractive as they are completely noninvasive, without requiring contact of any material to the wound site. The FLIR ONE thermal camera, used as an attachment to a smartphone, allowed for the assessment of healthy and damaged tissue in patients with diabetic foot ulcers.^157^ Analytical models were used to extract *k* from the infrared images. The authors detected temperature changes of up to 6.9 °C between the wound and adjacent tissue and associated reductions in *k* of up to 84.3%.^157^ While imaging techniques are clearly attractive for a multitude of applications, there are drawbacks related to cost and the requirement of patient immobilization for accurate measurements.^76^

**FIG. 11.**
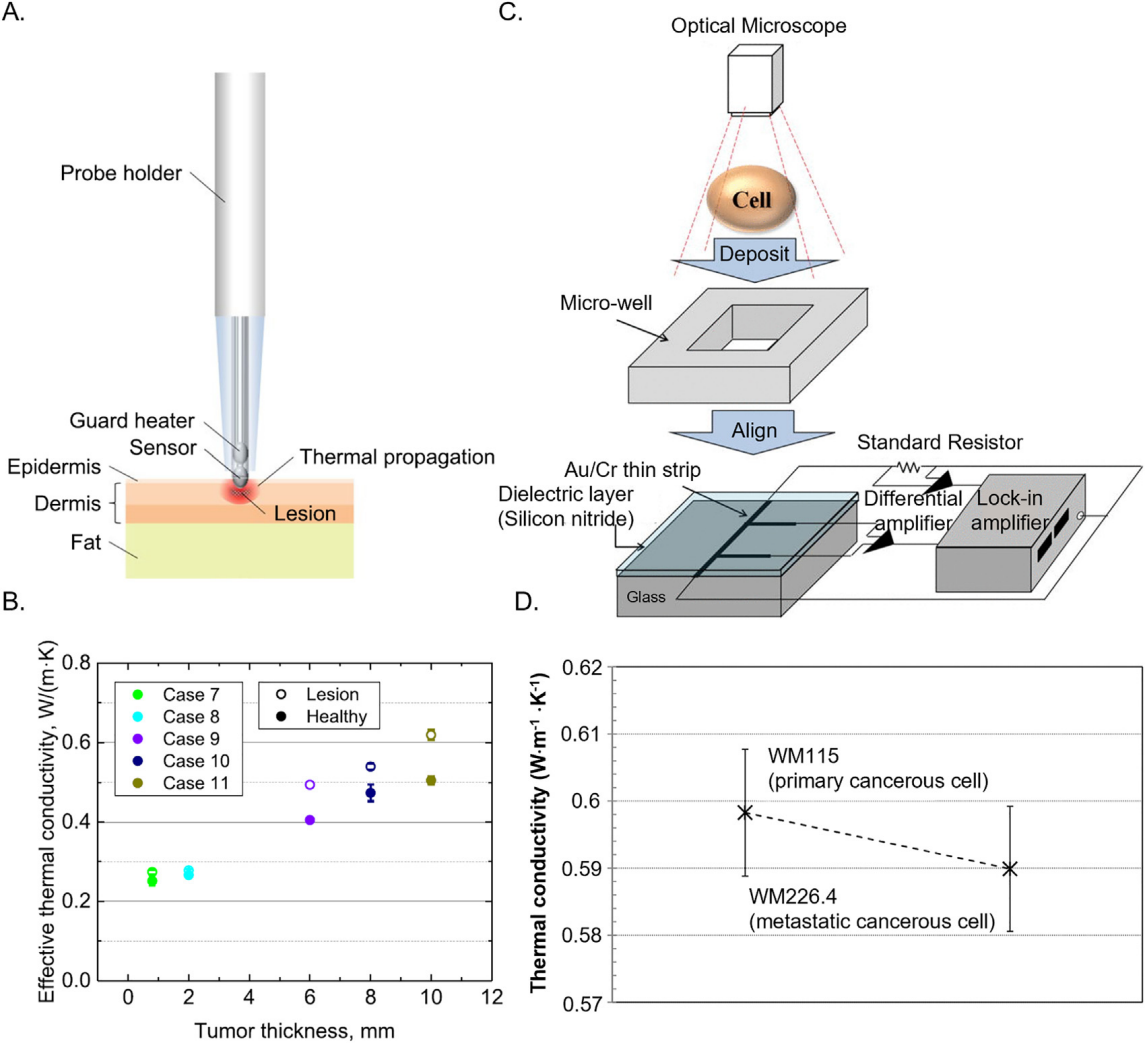
Sensing thermal transport properties for applications in skin cancer. (a) Schematic image of a guard heater probe in the form of a pen for applications in diagnosis and tracking of melanoma. The images are accompanied by **(**b) data on the measured effective k of the lesion and healthy regions of skin as a function of tumor thickness for five different patients. (c) Schematic image of the fabrication scheme for a microwell laying on top of a nanoscale heater for measurement of k of single cells accompanied by **(**d) data showing k for normal and cancerous cells. (a) and (b) Reproduced with permission from Okabe *et al.*, Sci. Rep. **9**, 3853 (2019). Copyright 2019 Authors, licensed under a Creative Commons Attribution (CC BY) license. (c) and (d) Reproduced with permission from Park *et al.*, J. Appl. Phys. **119**, 224701 (2016). Copyright 2016 AIP Publishing.

Okabe *et al.*'s work on a guard-heater thermistor probe in a pen-like format for characterization of malignant melanoma lesions represent another example of thermal sensing as the basis for assessing the health of the skin [Fig. 11(a)].^88^ The device utilizes a pulsed power decay technique (similar to the TPS method) whereby a constant DC voltage (initial power 3 mW) delivers thermal power in the form of a pulse (∼3 s duration) to the surface of the skin.^88^ Recordings of the temperature and power during heating and the temperature during cooling occur continuously, to allow determination of *k* through an energy balance equation. The result depends on the integral of the temporal change in temperature during cooling and the integral of the power across the thermistor during heating.^142^ The authors demonstrate the use of this scheme to show that the effective *k* of melanoma lesions are consistently higher than those of non-lesional areas [Fig. 11(b)]. In addition, the data indicate a positive, linear relationship between tumor thickness and *k*. The findings suggest a fast, simple, and noninvasive basis for characterizing melanoma tumors, with the potential to avoid cumbersome imaging techniques or invasive skin biopsies.

Through schemes in adjusting the measurement depth with the operating frequency, the 3ω method can determine the *k* of single, cancerous biological cells with volumes as small as ∼10 pl, where the effective depth is less than 10 *μ*m.^158^ In this setup, a nanoscale heater (half-width = 200 nm, allowing *h* < 5 *μ*m) defined using electron-beam lithography rests below a micro-well that confines a cell [Fig. 11(c)].^159^ Studies demonstrate that normal human skin cells (*k* = 0.593 W m^−1^ K^−1^) have *k* 2.2% higher than that of basal cell carcinoma cells (*k* = 0.579 W m^−1^ K^−1^) with high significance (p = 0.0031) [Fig. 11(d)]. Possible explanations for the difference in *k* between cancerous and normal skin cells are increased protein content in cancer cells, reduced water content in cancer cells, and an increase in glycogen content in conjunction with an increase in lipid content.^159^

Tian *et al.* report soft, skin-conformal 3ω sensors for detecting the thermal properties of the skin, with capabilities in accurate measurements of subtle changes in *k* and *α* of the skin, with the ability to adjust the effective depth of sensing through the operating frequency.^74^ This platform can monitor subtle changes in perfusion, for instance, through slap-induced hyperemia. Increased surface perfusion leads to increases in the thermal conductivity and diffusivity by ∼32% and ∼13.3%, respectively, indicating effusion of water out of the skin cells of the epidermis and increased microvascular blood flow in the dermis, consistent with visual reddening of the skin [Fig. 6(c)].

**FIG. 12.**
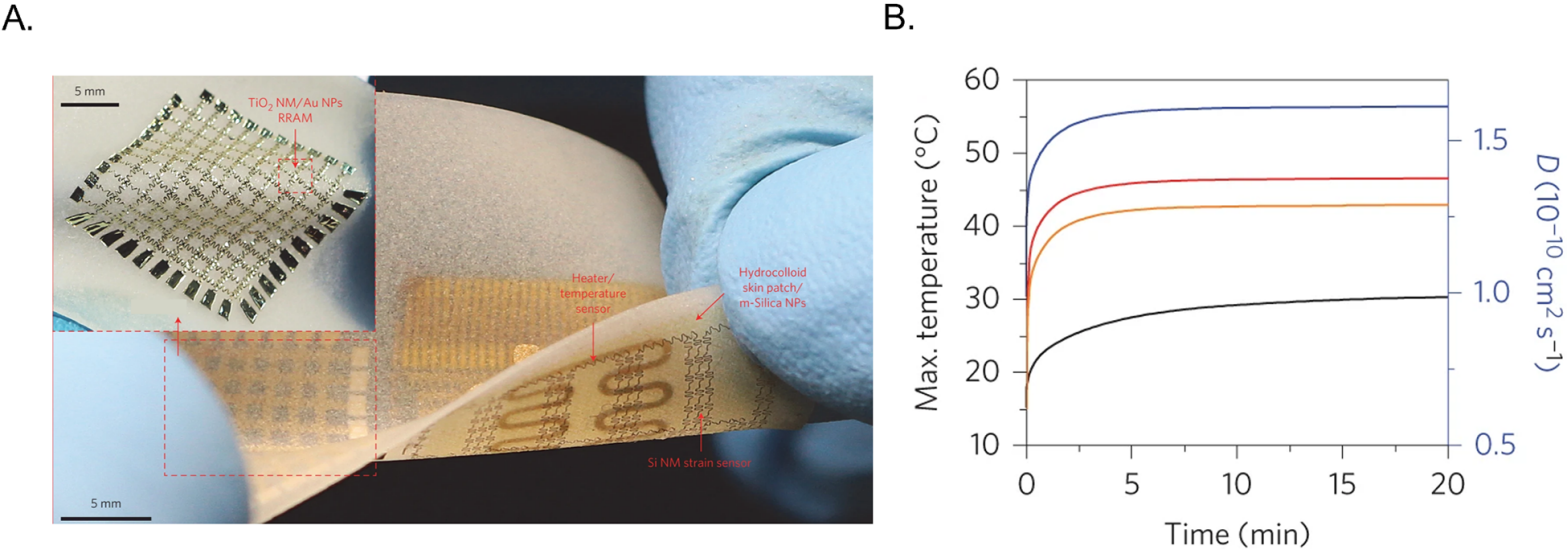
Simultaneous delivery of heat for therapy and sensing of the skin's thermal properties. (a) Photograph of a wearable memory patch containing an electro-resistive heater/temperature sensor. (b) Maximum temperature as a function of time on the heater surface (red), skin/patch interface (orange), and on the interface without heating (black). The blue curve represents the diffusion coefficient D of a therapeutic drug into the dermis with application of heat. Reproduced with permission from Son *et al.*, Nat. Nanotechnol. **9**, 397–404 (2014). Copyright 2014 Springer Nature.

Son *et al.* demonstrate applications of heat therapy and associated increases in *k* and *α*, thereby further establishing the dependence of the thermal properties of the skin on physiological parameters, such as blood flow and water content. These studies utilize thin film metal Joule heaters operated at high thermal power densities to heat the skin to different values based on the thermal diffusivity of the underlying tissue.^160^ In this work, the authors develop a wearable integrated memory unit to store data and trigger therapy [Fig. 12(a)]. The heat from the heaters accelerates diffusion of drug-loaded m-silica nanoparticles by first degrading the physical bonding between the nanoparticles and the drugs and next, increasing the transdermal drug diffusion rate [Fig. 12(b)]. Skin temperature is monitored during the drug delivery process. Thus, heat therapy and closed-loop feedback represent attractive options that can be useful in the diagnosis, monitoring, and therapy of skin diseases and tracking skin health for a multitude of applications.

## V. TECHNIQUES FOR SENSING DYNAMIC FLUID FLOW IN THE SKIN

Section IV focuses on “static” conditions where convective fluid flow in the skin can be neglected entirely or can be considered time invariant. As discussed earlier, volumetric blood perfusion, *w* describes omni-directional capillary/volumetric blood flows through a volume of tissue. Unlike perfusion, blood flow velocity through a vein or artery is a vector quantity, with magnitude *v* (expressed in mm/s or ml/min). Assessments of this quantity can be important for evaluations of vascular and tissue health.^161–163^ The following presents a brief overview of traditional measurements of near-surface *w* and *v* in the skin.

Conventional, noninvasive flow sensors (e.g., strain gauge plethysmography) rely on mechanical effects to measure *v* over a wide range (7–268 ml/min) via changes in volume of a particular body location (typically the limbs) during periods of occlusion and release by one or more pressure cuffs. Such approaches are, however, cumbersome for continuous tracking of changes in flow and they are susceptible to motion artifacts related to respiratory mechanics (movements of the thoracic cage).^164–166^ Acoustic techniques, such as contrast-enhanced ultrasound imaging, can evaluate blood flow/perfusion through the skin and other organs.^167–169^ Contrast-enhanced ultrasound is similar to conventional Doppler ultrasound, but with the addition of intravenous injection of contrast agents that perfuse through the bloodstream to other blood vessels and organs. The contrast agents are 1 – 7 *μ*m diameter microbubbles of air or perfluoro gas encased in a phospholipid, surfactant, or denatured albumin shell, designed to exhibit resonant frequencies in the range of 2–17 MHz, aligned with ultrasound used in clinical environments.^170^ Upon exposure to the ultrasound, the microbubbles oscillate, causing echo-enhancement and increased contrast.^168,170–172^ Continuous data cannot be obtained with this technique, however, due to the need for patient immobilization and intake of contrast agents for quantifying *w* and *v.*^171–174^

Optical sensing methods, such as laser doppler flowmetry (LDF) and photoplethysmography (PPG), avoid some of these limitations. LDF operates by illuminating a local surface area of tissue with a single wavelength of light (most commercial systems use a 780 nm laser source) and evaluating the resulting frequency shift in the light backscattered by red blood cells.^175^ The measurement typically uses an optical fiber probe, placed in contact with the skin, to emit the source wavelength and receive the backscattered signal. The spacing between the emitting and receiving fibers, along with the source wavelength dictate the measurement depth: e.g., for a 780-nm source wavelength, LDF can probe the skin at a depth of ∼0.73 mm and volume of 2.7 mm^3^.^175^ High temporal resolution (milliseconds) allows for rapid, continuous measurements.^176–178^ The net flux of red blood cells (the product of the red blood cell concentration and *v*) is directly proportional to the magnitude of the wavelength shift, thereby yielding an estimate of microcirculatory blood flow in arbitrary units, not perfusion units.^179^ LDF is sensitive to a wide range of *w* (0–300 ml/min per 100 g tissue).^180–186^ Disadvantages include poor spatial resolution and measurement artifacts related to deformation of the probe fibers and pressure applied to the skin by the probe.^187^ Laser speckle contrast imaging (LCSI) is a similar alternative to LDF, with the added advantage of large surface-area measurements.^188^ A red- or near-infrared laser source that illuminates the surface of tissue generates a random speckle pattern (varying spatial intensity) that is detected by a silicon charge coupled detector (CCD).^189^ In the presence of flow, the speckle pattern is “blurred” resulting in reduced contrast in the corresponding region of the image. The degree of contrast reduction depends on the blood velocity.^189^ While this technique does not require physical contact, the required optics cannot be integrated into a compact wearable device.

PPG relies on similar underlying measurement physics. An infrared (800–960 nm) LED is the most common illumination source, selected for absorption of hemoglobin in this range of wavelengths.^190^ A photodiode captures some of the backscattered light. PPG is most commonly used to measure heart rate, as the AC component of the backscattered light relates to pulsatile flow through the arterioles and arteries, independent of source wavelength.^191^ In contrast, the DC component of this signal represents non-pulsatile, average blood volume in the tissue at depths up to 3 mm, depending on the illumination wavelength and the distance between the LED and photodiode.^165,191–194^ Like LDF, PPG only yields qualitative estimates of *w*. Cycles of respiration, processes associated with thermoregulation, activity of the sympathetic nervous system, and overall motions of the body also affect the DC component of the PPG signal, as further complications to interpreting the response.^195^

Thermal sensors are attractive alternatives for noninvasive, quantitative measurements of both near-surface cutaneous *w* and *v*. These thermal flow sensors can easily be rendered into wearable and epidermal form factors, thereby removing measurement sensitivity to body motions (e.g., nonconformal contact/delamination of the sensor from the skin), the need for application of pressure to the skin (e.g., with a rigid probe), and bulky, wired electronics, allowing free motion of the user.^76,80,87,151,196^ Heaters and temperature sensors arranged in specific geometries allow for measurements of *v* at different depths into the tissue. For example, recent work illustrates a 4 × 4 array of small-area (1 mm^2^) thermal sensors for quantitative measurements of venous blood flow in the skin by way of multiple parameters—*w*, *v*, *k*, *α*, and *T*_0._^76^ Subsequent sections detail different thermal flow sensing techniques, followed by wearable architectures and their applications to skin and other important physiological health conditions.

### A. Measurements of perfusion

Thermal techniques to measure perfusion involve the same components as those for simple sensors of the skin's intrinsic thermal properties. A heater locally induces a temperature change (Δ*T*) on the surface of the skin, and one or more temperature sensors capture the resultant spatiotemporal changes in temperature. The main differences lie in analysis approaches and models of the transient and steady state thermal responses.^90^ Valvano *et al.* (Ref. 210) used an electrically resistive, spherical thermistor mounted at the tip of a needle, or catheter for measurements of *w* in a scheme known as the step-temperature technique, where (i) the initial temperature of the tissue *T*_0_ is measured and (ii) the thermistor is self-heated to a fixed, pre-determined temperature *T* > *T*_0_ while the power through the thermistor *q* is varied to maintain the constant *T*, for a measurement time on the order of ∼10–20 s, depending on the geometry of the probe.^90,197^ The thermal power *q* required to maintain *T* varies according to^90^

q(t)=Γ+Bf(t), (34)

where *Γ* is the steady state, volume-average power (W/ml) of the thermistor, *B* is the slope of the transient power rise (in W s^−1/2^/ml), and *f*(*t*) is the transient power function given by^90^

$$f\left(t\right)=\frac{e^{\frac{-wc_{\mathrm{bl}}\alpha_{m}}{k_{m}}t}}{\sqrt{t}}-\sqrt{\frac{-wc_{\mathrm{bl}}\alpha_{m}\pi }{k_{m}}}\mathrm{erfc}\sqrt{\frac{-wc_{\mathrm{bl}}\alpha_{m}}{k_{m}}t},$$ (35)

where *c*_bl_ is the specific heat capacity of blood, and *k*_m_ and *α*_m_ are the thermal conductivity and diffusivity of unperfused tissue matrix, respectively. An effective thermal conductivity (*k*_eff_) and diffusivity (*α*_eff_) model can be used to determine perfusion. The effective values combine the contributions of perfused and unperfused tissue. Equations (34) and (35) can be used to find the relationship between *k*_eff_ and *q*, through a closed solution to the well-known bioheat equation.^90^ The value of *k*_eff_ is^90^

$$k_{\mathrm{eff}}=\frac{1}{\frac{3T}{\Gamma a^{2}}-\frac{1}{5k_{b}}}.$$ (36)

*T* is the pre-determined temperature change during the measurement, *a* is the radius of the bead, and *k*_b_ is the thermal conductivity of the thermistor. When *w* = 0, *k*_eff_ = *k*_m_. The value of Γ can be determined by a linear regression of values of *f*(*t*) as a function of *t*^−1/2^. The value of *α*_eff_ is derived from the solution of the transient model and is given by^90^

$$\alpha {}_{\mathrm{eff}}={\left[\frac{a}{\sqrt{\pi }B/\Gamma \left(1+\frac{k_{m}}{5k_{b}}\right)}\right]}^{2}.$$ (37)

B/Γ can be computed from least squares regression of the measured values fit to (Γ+*Bt*^−1/2^), where *B* is the slope of the transient power curve. Perfusion can be isolated for the steady-state solution:^90^

$$w=\frac{{\left(k{}_{\mathrm{eff}}-k{}_{m}\right)}^{2}}{k_{m}c_{bl}a^{2}},$$ (38)

where *w* can also be calculated from the transient solution (*w*_2_):^90^

$$w_{2}=\frac{\sqrt{\alpha_{\mathrm{eff}}}-\sqrt{\alpha_{m}}}{\alpha_{m}c_{\mathrm{bl}}a^{2}}\;{\left[1+\frac{k_{m}}{5k_{b}}\right]}^{2}k_{m}.$$ (39)

Reference 90 provides a measurement procedure for calculating *w* with this model. With this approach, reproducibility in *k*_eff_ is better than 0.6% and that for *α*_eff_ is better than 2%, resulting in a 5% reproducibility in computed *w*. Systematic studies show that the measurement error for *w* from the *k*_eff_ model [Eq. (38)] determined by comparing true (acquired using radioactive tracing techniques) and computed values with tissue samples was 10.6%, while that from *α*_eff_ [Eq. (39)] was 24%. In addition, the sensitivity to changes in perfusion was ∼7% using Eq. (38). The use of *α*_eff_ to determine tissue perfusion, thus, requires further study.

An alternative approach known as the pulsed decay technique can also be used to measure *w*. Here, a short current pulse of constant *q* is applied to a thermistor or RTD.^197^ Δ*T* rises to a maximum value and then decays at a rate dependent on *w*, the geometry of the heating/sensing element and other factors. The analytical solution for the temperature decay at the center of a sphere is given by^197^

$$\Delta T=\frac{qa^{2}}{k}\left[\mathrm{erf}\left(\frac{1}{2\sqrt{z}}\right)-\frac{1}{\sqrt{\pi \tau }}e^{-\frac{1}{4\tau }}\right]e^{-\gamma \tau },$$ (40)

where *z* is the axial variable in meters, *τ* is dimensionless time, given by $\tau =\alpha t/a^{2}$, and *γ* is dimensionless blood flow parameter given by $\gamma =wc_{bl}a^{2}/k$. The values *k* and *α* represent the thermal transport properties of the tissue. The measurement sensitivity depends on *w*, *k* and sensor geometry. This sensitivity is given by^197^

$$\frac{\Delta T_{\mathrm{unperfused}}\left(t\right)-\Delta T_{\mathrm{perfused}}(t)}{\Delta T_{\mathrm{unperfused}}(t)}=0.1,$$ (41)

where $\Delta T_{\mathrm{unperfused}}(t)$ is the response when *w* = 0, $\Delta T_{\mathrm{perfused}}(t)$ is the response when *w* > 0, for a time window *t*_w_ (the length of time where the pulsed decay response is largely influenced by perfusion). Equation (41) represents a 10% difference between $\Delta T_{\mathrm{unperfused}}(t)$ and $\Delta T_{\mathrm{perfused}}(t)$. For example, for tissue density *ρ* = 1000 kg/m^3^, *c*_bl_ = 4000 J/kg K, *k* = 0.5 W/m K, the time window for a spherical probe of *r* = 3 mm and *w* = 10 kg/m^3^ s do detect an appreciable difference is 30 s, but for *r* = 6 mm and same *w* would be hundreds.^197^ The smallest measurable *w* (*w*_min_) depends on the sensor size. For a sensor with *r* = 2 mm, *w*_min_ = 30 kg/m^3^ s and for one with *r* = 8.6 mm, *w*_min_ = 1.6 kg/m^3^ s.^197^ Lower values of *w* require larger heating elements, a trend that applies to most heated thermistor approaches, including the step-temperature and constant power measurement schemes.

The closed analytical solutions described here and in Ref. 90 do not include tissue inhomogeneities due to large blood vessels or due to local variations in perfusion and tissue properties. In cases where such effects are significant, other analytical or numerical approaches may be required.^197^ Apart from these step-temperature and pulsed-decay techniques, other thermal methods for the measurement of perfusion include the heat-washout method (described in a later section)^198,199^ and combined heat flux/temperature sensor measurements.^200,201^

### B. Thermal sensing techniques for the measurement of anisotropic flow

**FIG. 13.**
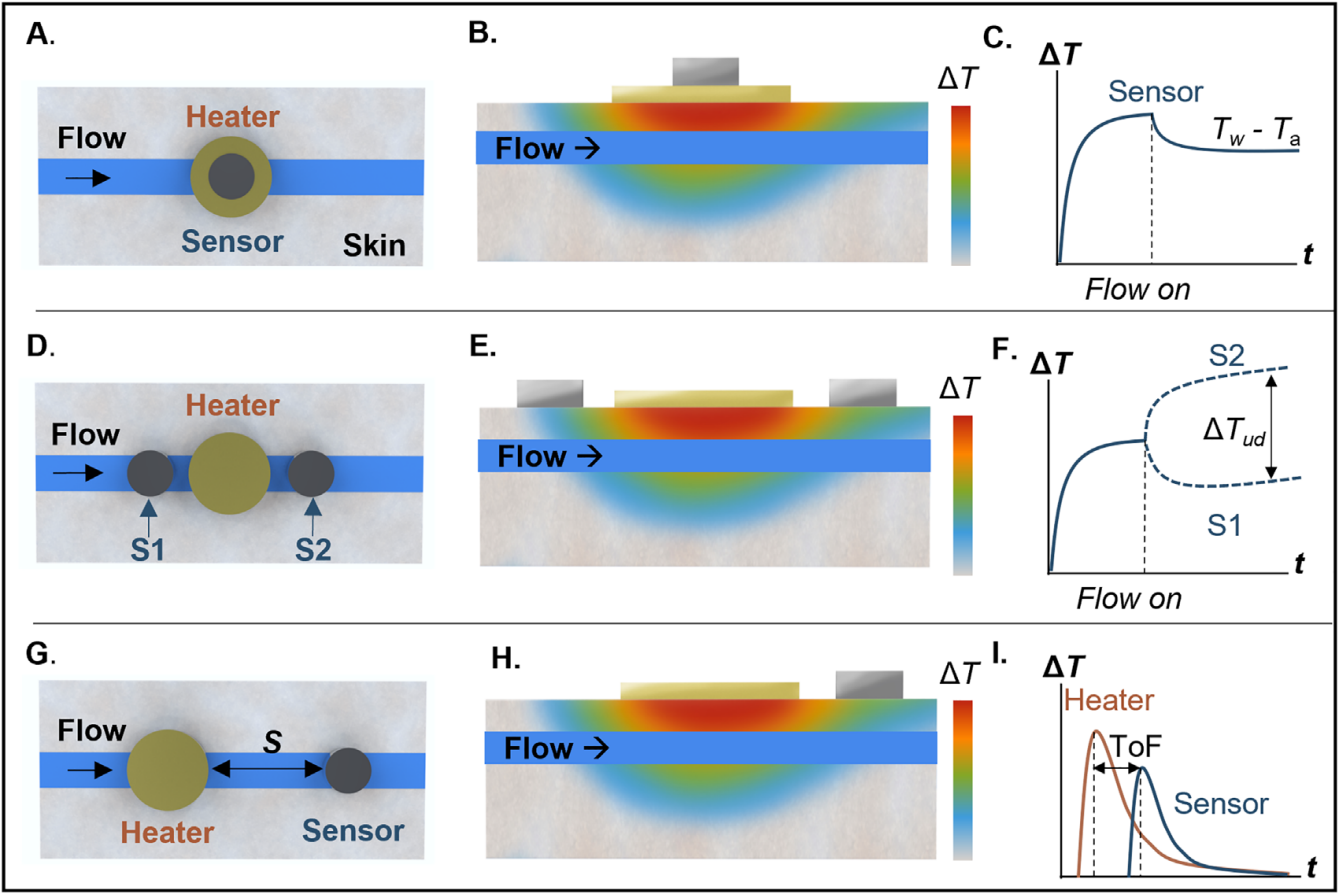
Thermal transport sending techniques for anisotropic flow. (a) Top view illustration of a thermal anemometry sensor overlaying a near-surface flow channel (e.g., blood vessel, subcutaneous catheter) in the skin. The heater (yellow) and sensor (gray) are thermally coupled elements. (b) A cross-sectional illustration of the anemometric sensor depicting the spread of heat (corresponding temperature change ΔT) at a particular timepoint when the heater is turned on. The color bar indicates the relative magnitude of ΔT with respect to depth into the skin. (c) Measurement of T_w_ – T_a_ as a function of time t with and without the introduction of fluid flow. T_w_ is the temperature of the heater and T_a_ is the ambient temperature (d) Top view illustration of a calorimetric sensor, consisting of a central heater and two temperature sensors placed upstream and downstream of the flow direction, separated from the heater by distances S1 and S2, respectively. (e) Cross sectional view of the calorimetric sensor and temperature profile at a particular time point when the heater is turned on (f) ΔT as a function of time as measured by the two sensors before (solid line) and after (dashed line) the presence of flow through the channel. The difference between the measured temperature of the upstream (S1) and downstream (S1) temperature is ΔT_ud_. (g) Top view illustration of a ToF sensor. A sensor is placed a distance S away from the heater downstream of the flow. (h) A cross-sectional view of the ToF sensor, analogous to (b) and (e). (i) Temporal variation of ΔT for both the heater and the sensor. The value of ToF, the “thermal time of flight” is the time-delay between the maxima of the ΔT of the sensor and the heater pulse.

In addition to measurements of non-directional, microvascular flow, determining the flow velocity through large vessels is of great importance. Macrovascular flow sensors also rely on a heater and one or more temperature sensors distributed along the flow channel. Figure 13 illustrates heater/sensor geometries for three thermal flow sensing techniques: (i) anemometry, (ii) calorimetry, and (iii) time-of-flight (ToF). In each of these methods, the heater operates in one of two modes: (i) to deliver constant heating power *q*, with variable temperature at the heater *T*_heater_ or (ii) to ensure a constant *T*_heater_, with variable *q*.

#### 1. Thermal anemometry

**FIG. 14.**
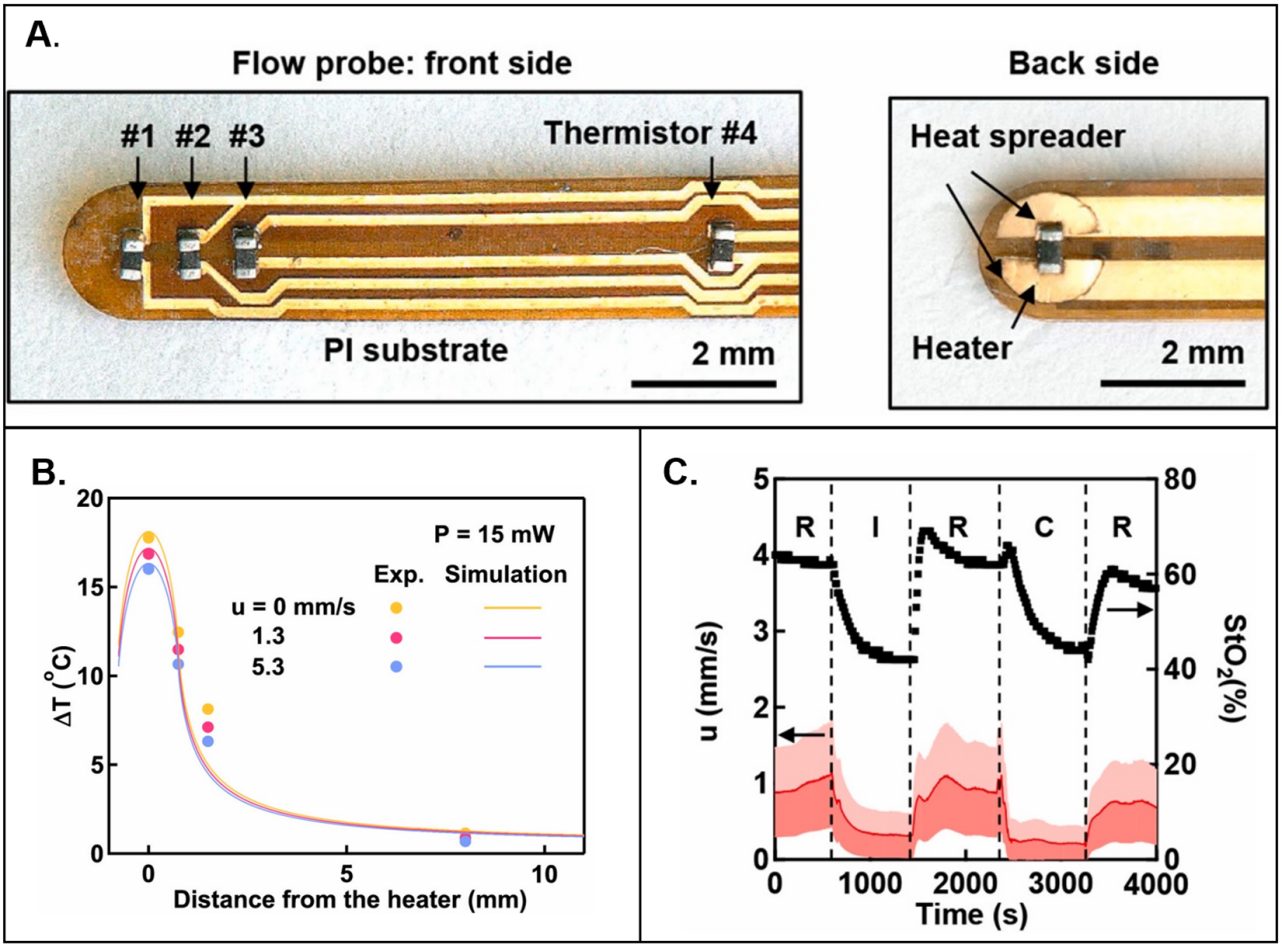
Implantable probe for the measurement of skin flap perfusion using thermal anemometry. (a) Photograph of the front-side (left) and back-side (right) of an implantable probe for measuring the perfusion of skin flaps using thermal anemometry. The device features a heater surrounded by a copper heat spreader, and four temperature sensors placed on the top-side of the probe, one directly above the heater and four at different distances away. (b) Measured ΔT from the four different temperature sensors for different microvascular flow rates, u. (c) *In vivo* microvascular flow rate as a function of time from a porcine skin flap during reperfusion (R), induced Ischemia (I), and induced Congestion (C). Tissue oxygen saturation (StO_2_) from a reference sensor is also plotted as a function of time. Reproduced with permission from Lu *et al.*, Biosens. Bioelectron. **206**, 114145 (2022). Copyright 2022 Elsevier.

Anemometric sensors offer the simplest geometry for the measurement of *v*, comprised of a single heating/sensing structure (the heater and sensor can be the same element, or two thermally coupled elements) [Figs. 13(a)–13(c)]. Most thermal anemometers use a single fine, cylindrical wire (∼1–15 *μ*m diameter) as both the heater and sensor^202^ for measurements over a wide range of *v* (< 2 *μ*l/min–10 l/min), with low noise (<0.01% of the mean value) and high-frequency responses (>100 kHz possible).^202–205^ The heater typically operates in a constant *T* mode, though operation in constant *q* mode is also possible. For the former, the current is adjusted, using sensing circuitry, to maintain a constant *T*_heater_. Underlying fluid flow causes convective heat loss.^202,206^ The resulting change in voltage *V* required to maintain constant *T*_heater_ can be used to determine *v*. The relationship between *V* and *v* for a hot wire anemometer can be analytically represented with a combined form of King's Law:^207,208^

$$V^{2}=k\left(A+B{\left(\frac{v}{\mu }\right)}^{n}\right)(T_{w}-T_{a}),$$ (42)

where *μ* and *k* are the fluid viscosity and thermal conductivity, respectively; *T*_w_ is the temperature of the wire; and *T*_a_ is the ambient fluid temperature. *A*, *B*, and *n* are empirical constants that depend on the sensor dimensions and fluid properties and can be obtained through calibration with known materials and fluids.^207,209^ In constant *T* mode, the expression (*T*_w_ – *T*_a_) is constant and *V* is a function of *v* alone, while in constant *q* mode, *V* is constant, and *T*_w_ is a function of *v*. Measurement accuracy of *v* using hot-wire anemometry can be affected by calibration accuracy and drifts in the values of *A*, *B*, and *n* in Eq. (42) related to change in fluid temperature and pressure.^210–213^ The above analysis is valid for anemometers embedded within the flow channel, typically for implanted devices in arteries, such as the aortic artery,^214^ or for the measurement of skin flap viability.^215^ The authors in Ref. 215 demonstrate a small probe (smaller than a 2-mm diameter biopsy needle) containing a heater, and four temperature sensors (one thermally coupled to the heater, similar to anemometry) for the measurement of microvascular flow velocity through skin flaps in porcine models **[**Figs. 14(a) and 14(b)]. This sensor operates in constant *q* mode, where a stable baseline *T*_w_ – *T*_a_ is achieved before inducing ischemia (clamping of the deep superior epigastric artery), reperfusion (release of the clamp), and congestion (clamping of the deep and superficial superior epigastric veins) of the flap [Fig. 14(c)]. The convective fluid flow changes the magnitude of *T*_w_ – *T*_a_ according to

$$\Delta T\;\approx \frac{qr}{k}\left(\frac{1}{1+0.76\chi \sqrt{\frac{vr}{\alpha_{\mathrm{fluid}}}}}\right)F\left(\frac{x}{r}\right),$$ (43)

where *r* is the circular radius of applied heat (using a copper ring heat spreader to improve the overall device sensitivity), χ is the volume fraction of blood, and *x* is the distance to the center of the heater, and $F\left(\frac{x}{r}\right)$ is

$$F\left(\frac{x}{r}\right)=\int_{0}^{\infty }J_{0}\left(\lambda x\right)J_{1}\left(\lambda r\right)\frac{d\lambda }{\lambda }.$$ (44)

*J*_0_ and *J*_1_ are the Bessel functions of the first kind (of orders 0 and 1, respectively). This model accounts for both convection and heat conduction. The relationship between *w* and *v* in the model presented in this work is given by

$$w=\frac{vA{}_{\mathrm{vessels}}}{\mathrm{Skin}\;\mathrm{Flap}\;\mathrm{Volume}},$$ (45)

where *A*_vessels_ is the effective, cross-sectional area of the microvascular blood vessels. Using these models, the authors find that the induced ischemia leads to a measured *v* = 0.3 mm/s, the congestive state causes *v* = 0.2 mm/s, for a baseline of *v* = 1 mm/s. Perfusion can also be computed using Eq. (45). Thermal anemometry, as demonstrated in this work, is promising for applications such as assessments of skin flap viability and in the future could be used for direct interfacing with large vessels of interest to determine macrovascular *v*. Development of wearable, noninvasive thermal anemometry devices is the subject of on-going work.

#### 2. Thermal calorimetry

**FIG. 15.**
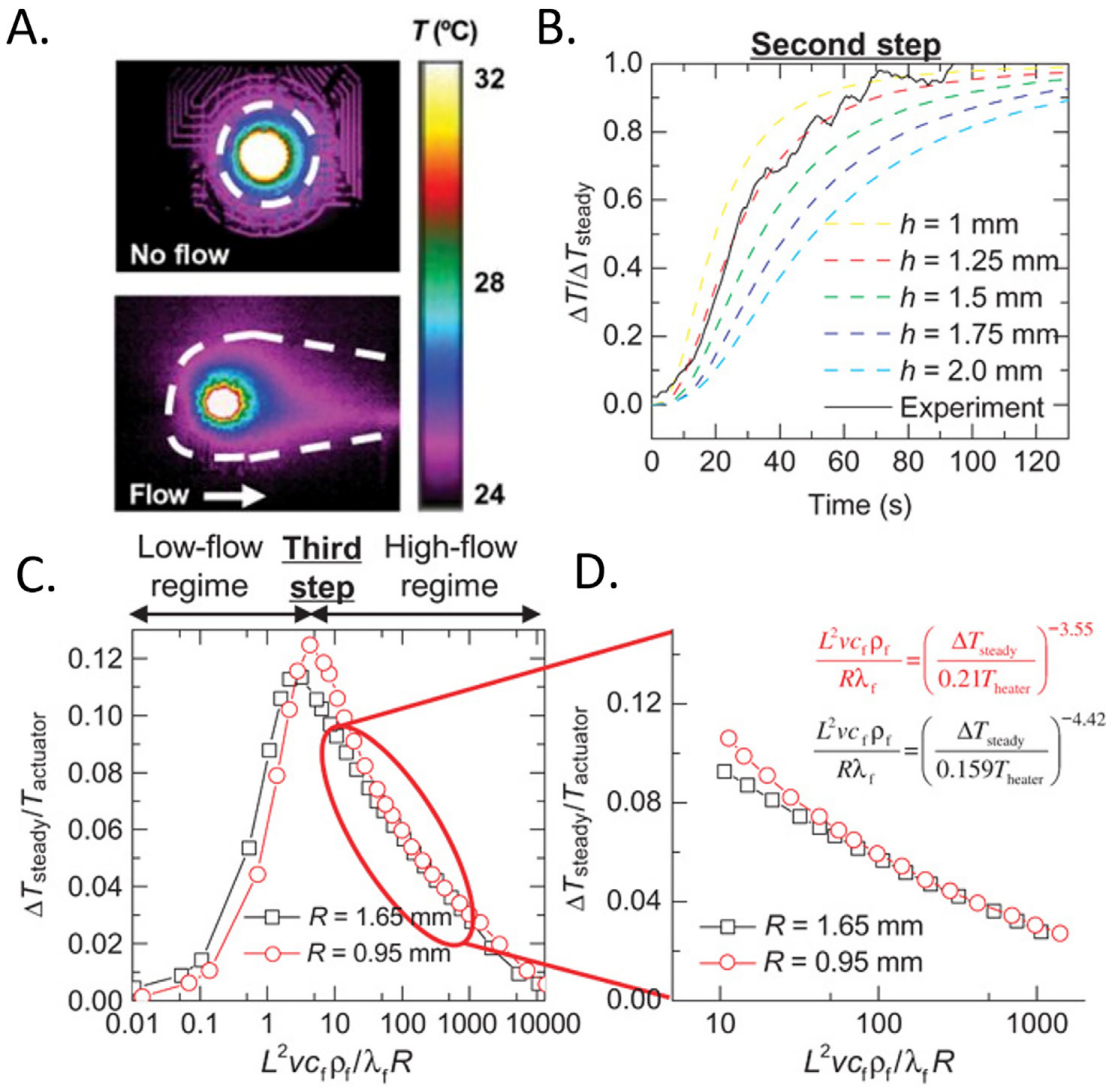
Calorimetric flow sensors for measurements of near-surface macrovascular blood flow. (a) Infrared camera image of a heater mounted on an artificial model of skin and a near-surface blood vessel, illustrating thermal anisotropy in the presence of flow. (b) Differential temperature between the upstream and downstream temperature sensors ΔT_ud_ normalized by its steady state value. The graph shows the experimental data and different curves of the same generated by finite element analysis to fit an accurate value of vessel depth (denoted here as h) beneath the skin surface. (c) Scaling law and FEA-generated curve showing the relationship between ΔT_ud_ normalized by ΔT_heater_ and several thermophysical parameters specific to the skin and sensor geometry for two different vessel radii. (d) An expanded view of the high-flow regime from (c) illustrating a monotonic relationship for relevant venous blood flow velocities and the sensor geometry from this reference. (a) Reproduced with permission from Krishnan *et al.*, Sci. Transl. Med. **10**, 465 (2018). Copyright 2018 AAAS. (b)–(d) Reproduced with permission from Webb *et al.*, Sci. Adv. **1**, 9, (2015). Copyright 2015 AAAS.

Calorimetric sensors use the constant *q* method. A thermal power density *q* induces a local temperature change (Δ*T*_heater_) on the surface of the skin, above the flow channel. Two temperature sensors, placed away from the heater at lateral distances *s*_1_ upstream of the flow and *s*_2_ (distance between the edge of the heater to the center of the temperature sensor) downstream of the flow, record a differential temperature Δ*T*_ud_ = Δ*T*_s1_ − Δ*T*_s2_ [Figs. 13(d)–13(f)]. Typically, *s*_1_ = *s_2_ = s*. The magnitude of *v* influences Δ*T*_ud_. When *v* = 0, the lateral heat distribution on the skin is isotropic, and hence, Δ*T*_ud_ = 0. When *v* is finite, Δ*T*_downstream_ > Δ*T*_upstream_, and thus, Δ*T*_ud_ > 0 [Fig. 15(a)].

Apart from *v*, several thermophysical properties affect Δ*T*_ud,_ including *k,* heat capacity *c*, and density *ρ* of both blood and tissue, blood vessel depth, and radius *R*_vessel_. Device geometry (i.e., heater radius *r*_heater_ and *s*) also influences Δ*T*_ud_. For example, for the epidermal calorimetric sensing structure reported by Krishnan *et al.*, Δ*T*_ud_ is maximum for *v* = 0.1 ml/min when *s* ≈ 3 mm.^196^ The values for *k, c,* and *ρ* of blood are well known in literature and can be assumed as constants.^151^ The multi-parameter dependence of Δ*T*_ud_ on tissue thermal properties and vessel geometry complicates the extraction of *v*, but recent research demonstrates methods to reduce the number of unknowns. To determine the tissue thermal transport parameters, the authors in Ref. 151 used TPS at short measurement times (*t* = 2 s) to measure local tissue *k*, *α* with a radial array of 14 individual epidermal sensors, each with *r* = 0.5 mm.^151^ Experimentally measured Δ*T*_ud_ normalized by its steady state value ( $\bar{\Delta T_{ud}}$) is independent of *v* and *r*_vessel_, allowing for the extraction of blood vessel depth through fitting to computational results based on finite element analysis [Fig. 15(b)]. Finally, $\bar{\Delta T_{ud}}$/Δ*T*_heater_ yields a relationship with the remaining two unknown variables, *r*_vessel_ and *v*; *r*_vessel_ can be approximated by values found in the literature specific to body location, leaving *v* as the final unknown variable.

Calorimetric sensors exhibit a non-monotonic response to *v* [Figs. 15(c) and 15(d)]. As *v* increases from *v* = 0, thermal diffusion dominates the response and $\bar{\Delta T_{ud}}$ increases until *v* = *v*_to_ (turnover velocity), after which convective cooling of the heater causes a decrease in the measured $\bar{\Delta T_{ud}}$ (i.e., $\frac{d\bar{\Delta T_{ud}}}{dv}<0$).^216^ The response, then, can occur in either the low flow regime (*v* < *v*_to_) or the high flow regime (*v* > *v*_to_). Sensitivity to a particular range of *v* and *v*_to_ can be tuned by adjusting *r*_heater_ and *S.*^216^ Venous blood flow velocities fall within the high-flow regime reported for the sensing geometry in Ref. 151 (*r*_heater_ = 1.5 mm, *s* = 1 or 3 mm). Epidermal thermal flow sensors can measure changes in *v* that have frequencies < 0.1 Hz, defined by the response time of the sensor for these measurement depths (∼10 s).^151^

The main sources of error in these measurements result from changes in ambient conditions (ambient temperature, skin temperature, and air convection) and from imperfect alignment of the sensors above the flow channel of interest.^87,151^ Air convection can be minimized with the inclusion of small, sealed air gaps or thermally insulating polyurethane (PU) foams placed above the heating/sensing components.^79,87^ Reference 87 reports an order-of-magnitude improvement in SNR with the PU foam approach. Changes in ambient/skin temperature can be accounted for using pulsed sensor operation or separate temperature sensors for measurements of environmental/skin temperature.^151,156^

#### 3. Thermal time of flight

Calorimetric sensors can also be operated as thermal ToF sensors,^217^ also using the constant *q* method. The simplest layout comprises a heater and single temperature sensor (separated by distance *S*) overlaying the flow channel [Figs. 13(g)–13(i)]. The heater delivers a thermal pulse to the skin (typically < 100 mW, < 2 s duration). A downstream sensor captures Δ*T* as a function of *t* (transit time of the thermal pulse) [Figs. 7(h)–7(i)].^218,219^ Assuming a line heating source of length *L* (often used in ToF systems), heat transport through a fluid is given by the combined diffusion and forced convection equation as follows:^220^

$$\frac{\partial (\Delta T)}{\partial t}+v\nabla \left(\Delta T\right)=\frac{k}{\rho c}\nabla^{2}\left(\Delta T\right)+\frac{Q}{\rho c},$$ (46)

where *Q* is the total the heat (in Joules) injected into the system by the heater. The analytical solution to this equation is^221^

$$\Delta T\left(S,y,t\right)=\frac{q}{4\pi k}\text{exp}\left\{-\left(\frac{{\left(S-vt\right)}^{2}+y^{2}}{4\alpha t}\right)\right\}.$$ (47)

Differentiating Eq. (47) with respect to *t* at the location of the heater/sensor (*y* = 0) yields the “time of flight”(ToF) as follows:^220^

$$v=\frac{S}{\mathrm{ToF}}.$$ (48)

Equation (48) is valid at high *v*, for the condition 4*αt* ≪ S, corresponding to the case that forced convection from flow dominates the response and the influence from thermal diffusion is minimal.^220^ At low *v*, when diffusion must be considered, the following holds:^220^

$$\mathrm{ToF}=\left\{\begin{array}{ll} \frac{-2\alpha +{\left(4\alpha^{2}+v^{2}S^{2}\right)}^{\frac{1}{2}}}{v^{2}}, & v\neq 0,\\ \;\frac{S^{2}}{4\alpha }, & v=0. \end{array}\right.$$ (49)

The value of *τ* is experimentally derived from ${\left.\frac{d\left(\Delta T\right)}{dt}\right|}_{t=\mathrm{ToF}}=0$, which represents the maximum of the Δ*T* vs t curve measured by the downstream temperature sensor.^218^ ToF sensors are most suited for measuring low flow rates^222^ at small values of Δ*T* (∼1 °C).^219^ *τ* can be detected for these small ∼1 °C thermal pulses even in the presence of baseline *T* drifts (as high as ± 5 °C): evaluation of ${\left.\frac{d^{2}T}{dt^{2}}\right|}_{t={\mathrm{ToF}}_{2}}=0$ yields *τ*_2_ which has sensitivity only to the thermal pulse, and is a faster measure of pulse transit time.^219^

**FIG. 16.**
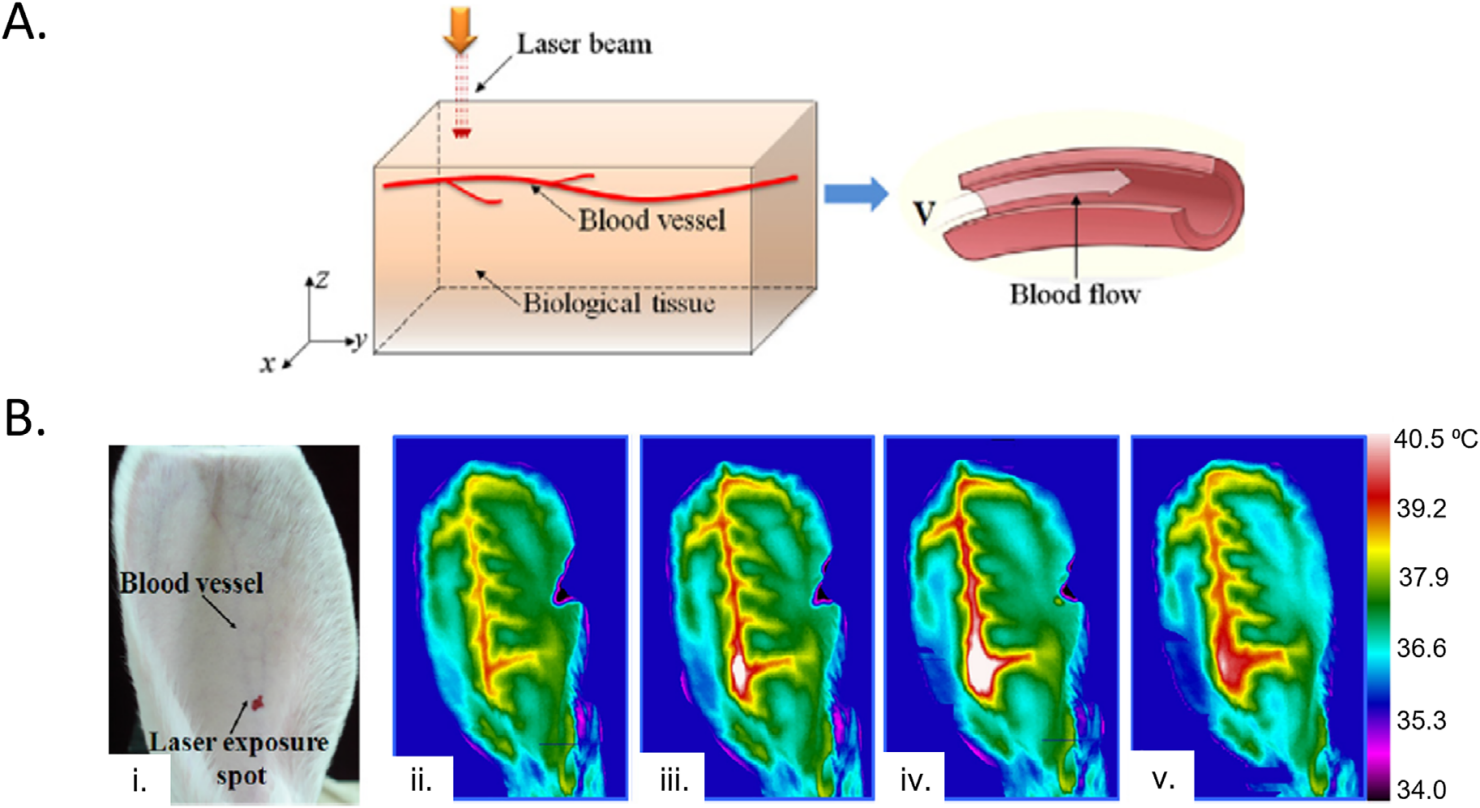
Laser-Induced Dynamic Thermography (LIDT**)**. (a) Schematic image of a laser beam incident upon skin with an underlying, superficial blood vessel for measurements of macrovascular flow velocity. (b) (i) Rabbit ear with marked locations of the blood vessel and laser spot exposure accompanied by infrared camera images of the rabbit ear (ii) before exposure to the laser, t = 0 s, (iii) 10 s after the start of laser heating, (iv) 30 s, corresponding to the maximum laser heating time, and (v) post-exposure at t = 50 s. Reproduced with permission from Lahiri *et al.*, Infrared Phys. Technol. **55**, 6 (2012). Copyright 2012 Elsevier.

Point measurements of temperature or temperature maps obtained by an infrared camera alone can be insufficient for characterizing complex health conditions, such as cardiovascular disease.^149^ Instead, parameters of vascular blood flow velocity, viscosity, volume, and pressure are of much greater relevance.^149^ Laser-induced dynamic thermography (LIDT) allows for the measurement of *v* in superficial veins/arteries by local heating of the skin using a laser source (810-nm, 1.2 W through an optical fiber of diameter 100 *μ*m) and tracking skin temperature changes with infrared thermography. Jin *et al.* demonstrated the use of LIDT to measure superficial arterial *v* in rabbit auricle skin.^149^ The optical fiber probe (placed 50 mm away from the rabbit ear) focused on a local area of the skin above the auricular artery and induced local heating (< 10 °C) for 30 s [Fig. 16(a)]. Infrared thermography (sampling rate of 3.75 frames/second) captured spatiotemporal characteristics of the heat before (10 s), during (30 s), and after (80 s) delivery of the heat to the skin. Similar to thermal ToF, *v* follows from measurements of the transit time *τ* across a given distance over the artery [Fig. 16(b)]. Post hoc image processing and analysis isolates the auricular artery from the rest of the image to ultimately determine *v* (36.94 and 38.74 mm/s for the left and right artery, respectively) [Fig. 9(a)]. Theoretical models that include the thermal properties of blood and tissue (*k*, mass *m*, and *c_p_*), the heat transfer coefficient *H* and temperature *T* of blood and air result in values that are comparable to those obtained by Doppler ultrasound. Errors in measurements of the temperature of the blood lead to ∼5% error in extracted *v*, and error in *H*_blood_, *H*_air_ leads to ∼6% and 22% error in *v*, respectively, illustrating that air convection can have a significant influence on the measurement. Uncertainty in the depth of the vessels beneath the skin surface represents an additional source of error in the computed *v*.

### C. Applications of wearable macrovascular flow sensors

Recent research in noninvasive and wearable thermal flow sensors create means for continuous monitoring of patient health, of direct clinical relevance. Applications range from mapping of micro- and macro-vascular cutaneous blood flow during various physiological circumstances to measurements of ventricular shunt flow in hydrocephalous patients through the skin.

Techniques based on thermal anemometry can measure cutaneous, microvascular *w* of the skin with peripheral neuropathy and atherosclerosis in type 2 diabetes patients.^198,199^ References 198 and 199 report an anemometer-based heat-washout method, performed using a probe consisting of a thermally coupled heater and temperature sensor attached to the skin using silk tape and contact fluid between the skin and probe.^198,199,223^ After measurement of the initial temperature of the skin *T*_0_, heating for 3–5 min with constant *q* yields a steady state increase in temperature $\bar{\Delta T}$ between 2 and 10 °C. After saturation at $\bar{\Delta T}$, the heater was turned off and skin *T* was recorded at a sampling rate of 0.1 Hz until saturating at ∼*T*_0_. The heat “washout” refers to the measured exponential temperature decay after the heater is turned off. Plotting Δ*T* as a function of *t* in logarithmic scale produces a straight line with a slope *m* (units min^−1^) proportional to *Q*^224^

$$Q=\frac{\Delta w}{\Delta V}=\frac{m}{K}\left(100\;\mathrm{ml}\;{\left(100\;g\cdot \text{min}\right)}^{-1}\right),$$ (50)

where Δ*w* is the local volumetric rate of blood flow leaving a volume of tissue Δ*V*. *K* is the tissue partition coefficient, given by

$$K=\frac{{\left(\rho c_{p}\right)}_{\mathrm{tissue}}}{{\left(\rho c_{p}\right)}_{\mathrm{blood}}}.$$ (51)

The authors in Ref. 224 assume constant *K* ≈ 1 ml g^−1^, based on literature values for the thermal properties of tissue and blood for all computations of *w*. The mean *w* for diabetic patients [47.6 ml (100 g min)^−1^] and healthy patients [53 ml (100 g min)^−1^], when lying in a recumbent position on the forefoot were comparable. However, differences arose during changes in posture. Lowering the leg 50 cm below heart level increased *w* of the right hallux by > 50% in 5/7 non-diabetic patients with intermittent claudication (a symptom of peripheral artery disease), where the remaining two experienced a slightly lower increase in *w*; this change was < 20% in 4/6 healthy patients, where the remaining two subjects exhibited no change in *w*. In diabetic patients, the increase in *w* was > 20% in 7/9 patients. Thermal flow sensing of this type, therefore, holds potential for identifying thermal signatures associated with atherosclerosis and peripheral neuropathy in the pathogenesis of diabetic foot ulcer formation.

**FIG. 17.**
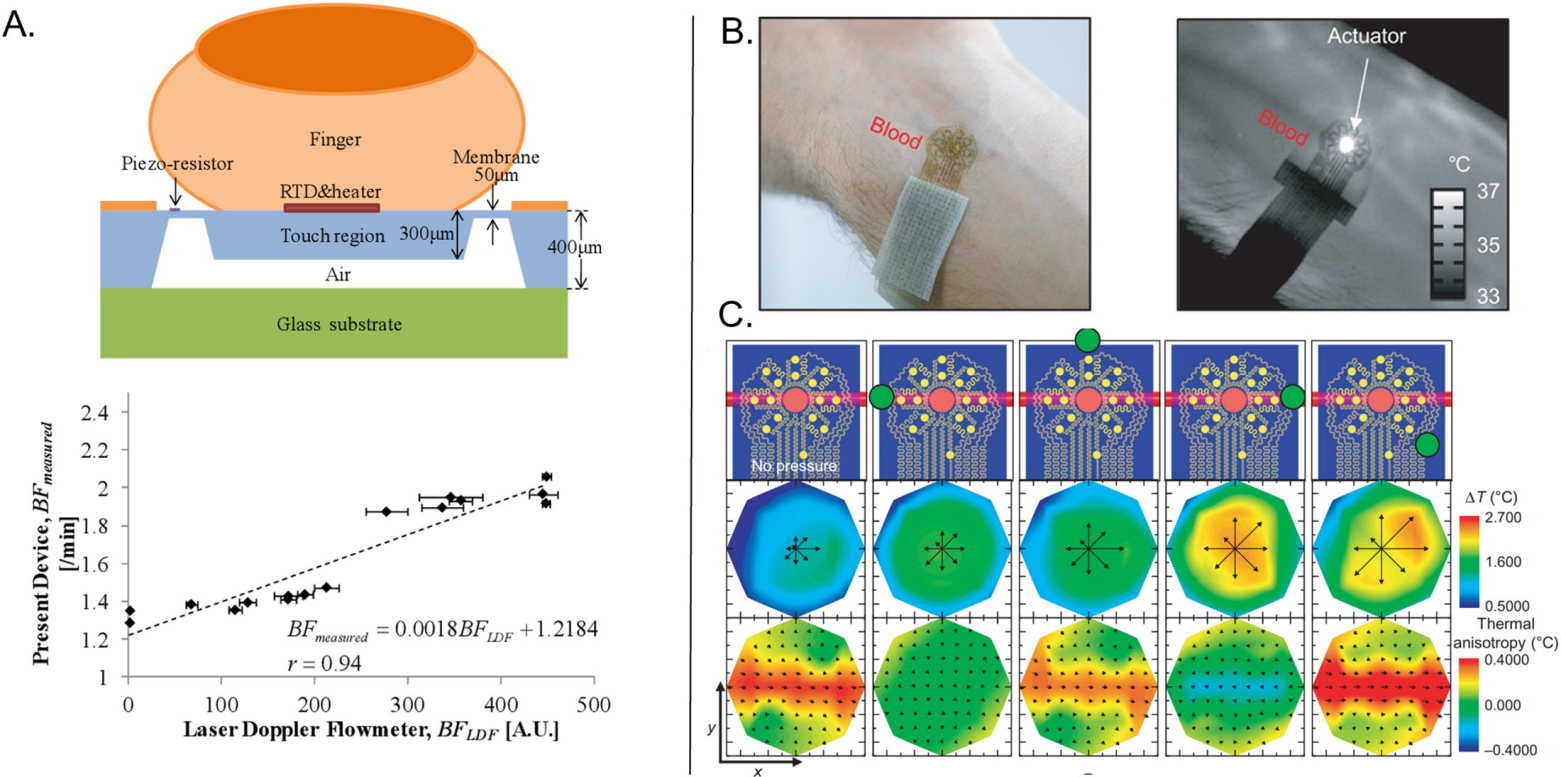
Thermal sensors for applications in assessing micro and macrovascular flow. (a) Schematic image of a finger mounted on a thermal flow sensor with contact force compensation for measurement of perfusion. The thermal flow sensor is comprised of a thin film RTD sensor which simultaneously serves as the heater. The measurement employs the heat-washout method. The contact force compensation is accomplished with four doped-Si piezo-resistors. The illustration is accompanied by measurements from the device in comparison with those from a commercial laser Doppler flowmeter. (b) Photograph and infrared camera image of a calorimetric flow sensor comprised of 14 temperature sensors surrounding a central heater. (c) Illustrations of the device overlaying a near-surface blood vessel and green dots indicating points of applied pressure surrounding the perimeter of the device. Below are the measured ΔT after heating for all 14 temperature sensors, followed by ΔT_ud_ from geometrically opposing pairs of sensors, indicating occlusion when pressure is applied to the location of the vessel, and the presence of flow in all other locations. (a) Reproduced with permission from Sim *et al.*, J. Micromech. Microeng. **22**, 125014 (2012). Copyright 2012 IOP Publishing. (b) and (c) Reproduced with permission from Webb *et al.*, Sci. Adv. **1**, e1500701 (2015). Copyright 2015, Authors, licensed under a Creative Commons Attribution (CC BY) license.

Sim *et al.* report a miniaturized device (20 × 23 × 0.9 mm^3^) using a similar heat-washout technique for the measurement of peripheral blood perfusion with skin contact force compensation [Fig. 17(a)].^225^ The device in this case consists of a thin-film (300-nm Ni) metal RTD and four doped-Si piezo resistors as a contact force sensor. The authors compare the performance of the heat washout data to perfusion units measured by LDF with linear correlation (*r* = 0.94). Large differences in measured blood flow from reference sites with no contact force applied are observed (up to ∼75%) when appreciable contact force (∼3N) is applied to the skin (inducing vasoconstriction). Experimental studies on human subjects using both the heat-washout sensor and LDF reveal an approximately linear dependence of the measured blood flow on contact force (− 31.7% N^−1^). Contact force compensation using this linear approximation reduces differences between measurement and reference sites to < 6.4%.

Soft, epidermal electronics for thermal calorimetry have also been used for measurements venous blood flow and tissue perfusion for applications in vascular diseases like atherosclerosis, sickle cell anemia, diabetes, and chronic kidney disease, and inflammation induced by external sources, such as sunburns [Fig. 17(b)].^151^ The device platform reported in this work allows for the detection of flow in vessels with a maximum depth of 2 mm and radius as small as 0.25 mm, and a flow rate range of *v* = 0.1–100 mm/s.^151^ The epidermal form factor allows for conformal contact, reducing error related to poor thermal contact and to relative motion between the device and skin. A radial array of ∼14 temperature sensors (*r*_sensor_ = 0.5 mm) surrounding a central heater (*r*_heater_ = 1.5 mm) at *s* = 3.5- or 5.5-mm heater-edge-to sensor-center spacing allows for spatial mapping of flow rate without the need for precise alignment over the superficial vein/artery of interest. Applying pressure to various locations around the perimeter of the device (laying over a near-surface vessel in the wrist) illustrates the ability to track both magnitude and direction of blood flow without exact placement. Pressure applied directly above the vessel reduces the measured blood flow, while application away from the vessel causes no appreciable change in the measurements. The same experiment performed over a region of the forearm with no visible large blood vessels illustrates no measurable change with applied pressure, indicating the ability to distinguish between regions of macrovascular and microvascular flow. Device measurements of venous blood flow overlying a vein in the wrist during occlusion/reperfusion of the upper arm by a blood pressure cuff (∼200 mm Hg) and comparison with an LCSI tool show good correlation. The epidermal calorimetry device captures additional pulsatory signals for a subject with deep veins (∼1.7 mm) where the LCSI tool does not, due to limited measurement depth of the latter. The detection of perfusion can be accomplished with measurements of Δ*T*_ud_ overlying regions, as described above, without the presence of large vessels, such as the fingertips. The authors demonstrate the ability to track perfusion changes, with good correlation to an LCSI tool, associated with induced urticaria and deep breathing.

**FIG. 18.**
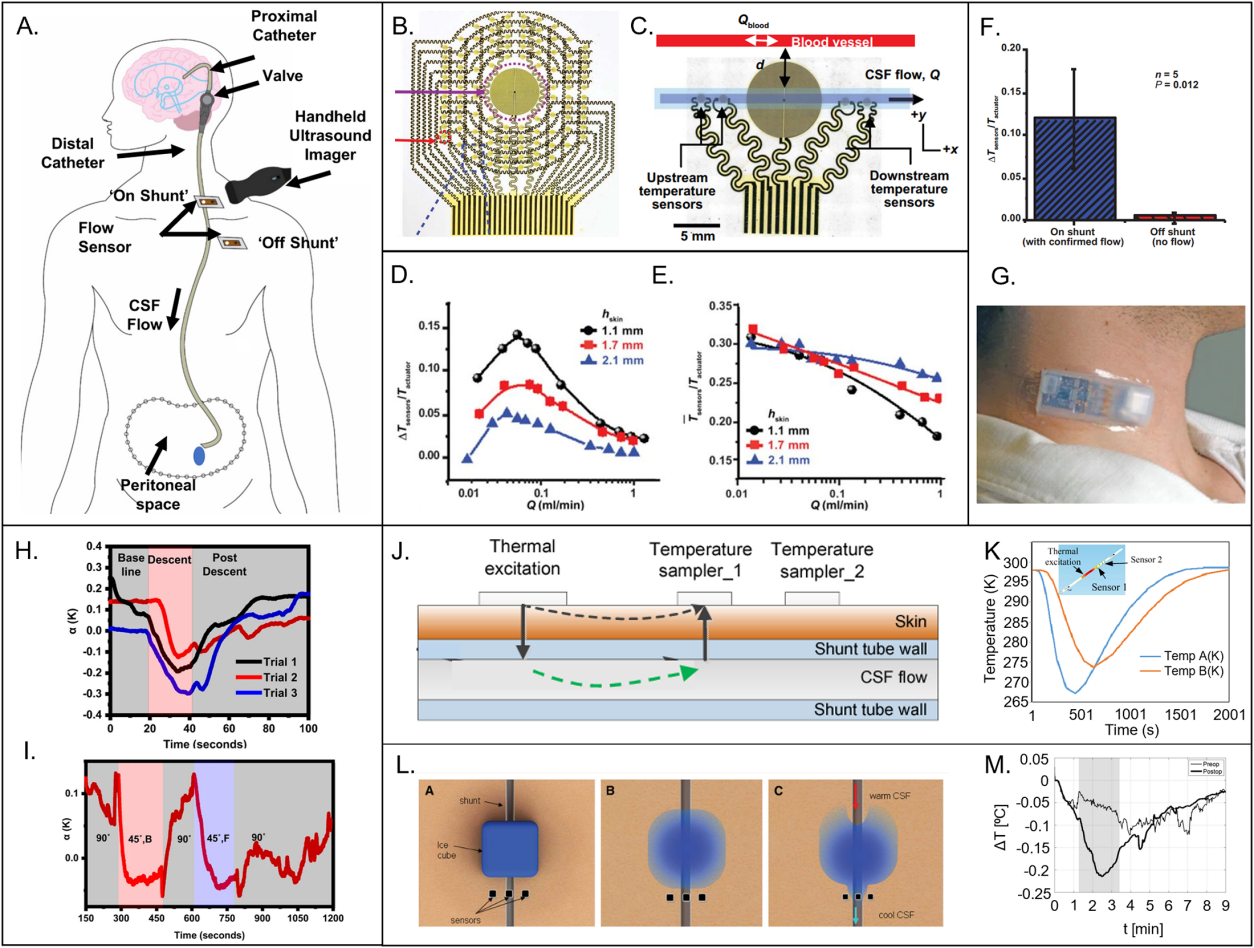
Thermal flow sensors for measurements of ventriculoperitoneal shunt flow. (a) Illustration of an implanted ventriculoperitoneal (VP) shunt for drainage of excess cerebrospinal fluid (CSF) into the peritoneal space. (b) Optical image of an epidermal square array for determining location of the implanted catheter and flow magnitude. (c) Optical image of an epidermal linear array for measuring flow magnitude through a catheter of known location. (d) ΔT_ud_ normalized by ΔT_heater_ and (e) the average temperature between upstream and downstream temperatures $\bar{\Delta T_{\mathrm{ud}\;}}\text{normalized}\;\text{by}\;\Delta T\text{heater}$ as a function of flow rate for different skin thicknesses, h_skin_. (f) ΔT_ud_/ΔT_heater_ for patients with confirmed CSF flow and no flow through implanted shunts. (g) Wireless embodiments of a calorimetric flow sensor which transfers data through Bluetooth Low Energy (BLE) protocols. (h) ΔT_ud_/ΔT_heater_ for a patient descending on an elevator moving at 1.5 m/s. (i) ΔT_ud_/ΔT_heater_ for a patient sitting up at 90° and leaning backward and forward at 45°. (j) Diagram of a thermoelectric time-of-flight device for sensing flow through VP shunts accompanied by (k) Data illustrating the temporal change in temperature for two sensors downstream of a thermoelectric cooling element. (l) Diagram of an ice cube placed on the skin and temperature sensors from the ShuntCheck device used to detect VP shunt flow using similar time-of-flight mechanisms. (m) ΔT as a function of t for a patient before and after corrective surgical intervention to restore shunt flow for a malfunctioning shunt. (a), (g), (h), and (i) Reproduced with permission from Krishnan *et al.*, NPJ Digital Med. **3**, 29, (2020). Copyright 2020 Authors, licensed under a Creative Commons Attribution (CC BY) license. (b)–(f) Reproduced with permission from Krishnan *et al.*, Sci. Transl. Med. **10**, 465 (2018). Copyright 2018 AAAS. (j) and (k) Reproduced with permission from Rajasekaran *et al.*, IEEE Sens. **2015**, 946–949 (2015). Copyright 2015 IEEE. (l)–(m) Reproduced with permission from Madsen *et al.*, Neurosurgery **68**, 198–205 (2011). Copyright 2011 Wolvers Kluwer.

Krishnan *et al.* followed up on the work of Ref. 151 for a different application: monitoring of ventricular shunt flow through the skin in hydrocephalous patients. Hydrocephalous is a neurological disorder characterized by overproduction or compromised reabsorption of cerebrospinal fluid (CSF) produced in the ventricular system of the brain.^226^ Treatment typically entails implantation of a thin silicone catheter (< 3 mm outer diameter) that drains CSF from the brain to a different part of the body, such as the peritoneum, pleural cavity, or right atrium. Malfunction of a valve (“shunt”) that regulates the flow rate of CSF or clogging of the catheter itself can lead to nonspecific clinical systems, thus typically requiring imaging (x-ray, CT, and MRI) for accurate diagnosis [Fig. 18(a)].^226–228^ The catheters pass through superficial layers of the clavicular dermis (1 – 2 mm) and support relatively low rates of flow of CSF (< 1 ml/min), ideal for noninvasive thermal sensing from the surface of the skin.^87,196,229^ Soft, epidermal devices designed for this purpose include (1) a square array of ∼100 temperature sensors surrounding a central heater (*r*_heater_ = 2.5 mm) [Fig. 18(b)] and (2) a linear array of 4 temperature sensors spaced equidistantly from the central heater (of same radius) [Fig. 18(c)]. The square array enables evaluation of catheter position and the presence of flow. The linear array represents a simplified form of the square array, for measurements of *v* when the catheter position is known. For a typical patient, the authors find that *v*_to_ = 0.05 ml/min, within the range of physiologically relevant CSF flow rates through the catheter [Fig. 18(d)]. In general, a measurement of Δ*T*_ud_ yields two solutions for *v*. To discern whether the measurement lies within the high- or low-flow regime, the authors propose use of the average of the upstream/downstream temperature sensors $\bar{T{}_{ud}}=\frac{1}{2}(T_{us}+T_{DS})$. *T*_DS_ varies non-monotonically with *v*, while *T*_US_ decreases monotonically for all *v*; hence, $\bar{T{}_{ud}}$ is nearly constant for *v* < *v*_to_ and decreases for *v* > *v*_to_ **[**Fig. 18(e)].^87^ Data on hydrocephalous patients indicate cases of occluded flow (*v* = 0.01–0.0027 ml/min) which are nearly one order of magnitude lower than those of un-occluded, healthy flow rates (*v* = 0.13–0.36 ml/min) with statistical significance (*p* = 0.012) [Fig. 18(f)]. Measurements of flow through the catheter before and after surgical revision and other treatments show trends that agree with clinical imaging (CT and MRI) and evaluations of patient health status. Demonstrations of wireless data transmission capability through BLE and a follow-up work (2020) allow for untethered/unrestricted patient movements and studies of flow rate with respect to patient posture [Figs. 18(g)–18(i)].^87^ The systems described in Ref. 87 use commercial SMD resistors and NTC thermistors for the heater/sensor, respectively, for ease of fabrication and reduction of overall costs.

Thermoelectric ToF has also been used to find CSF flow rates through hydrocephalous shunts.^222,230^ Instead of heating, local cooling of the skin (∼4° C) using a semiconductor thermoelectric cooling (TEC) device produces the required temperature gradient for the measurement [Fig. 18(j)]. Other work uses ice instead of a TEC for local cooling of the skin.^231,232^ The time-difference *τ* between the minimum temperature (resulting from the cooling) measured by the two temperature sensors (located at distances 20 and 30 mm downstream of the TEC, respectively) is the time of flight. The value of *v* is computed as the distance between the two temperature sensors (Δ*y*) divided by *τ*. The authors in Ref. 230 derive *v* through a thermal equivalent circuit (lumped element model) for the skin, CSF, and modified catheter tube including sections of titanium to enhance thermal coupling to the skin. The relevant parameters are *R*_l_, the lateral thermal resistance along the skin, *R*_sh_, that along the catheter tube, *R*_sk_, between the skin and titanium, and *R*_cv_, between the titanium and CSF. Replacing three sections, directly underlying the TEC and temperature sensors, of the silicone catheter tube with a biocompatible, high-thermal *k* metal (titanium, 21.9 W/m K) reduces *R*_sk_ and *R*_cv_. The authors illustrate benchtop sensing capabilities for physiologically relevant CSF flow velocities (*v* = 0.5–1 mm/s) and ∼1.2 mm thick skin [Fig. 18(k)].

In another measurement strategy, a commercial device called the “ShuntCheck” (similar to an anemometer) measures Δ*T* = $T{}_{\mathrm{center}}$ - ${\bar{T}}_{\mathrm{ref}}$ (induced by an ice cube applied to the skin for ∼60 s) from a central temperature sensor directly along the flow channel (*T*_center_) compared to the average of two reference temperature sensors ( ${\bar{T}}_{\mathrm{ref}}$) lying perpendicular to the direction of flow [Fig. 18(l)].^231^ In the presence of flow, the central temperature sensor experiences net cooler *T* than the reference temperature sensors; conversely, when there is no flow, all three temperature sensors record nearly the same *T*. Measurements on hydrocephalous patients with malfunctioning shunts pre-and post-operatively illustrate visible Δ*T* minima associated with the presence of flow and with good statistical sensitivity (80%) and specificity (100%) [Fig. 18(m)].

## VI. CONCLUSIONS AND OUTLOOK

Emerging methods in thermal sensing provide the basis for versatile measurements of skin health. The ability to capture spatiotemporal variations of temperature across the surface of the skin has widespread potential for monitoring skin disorders. Measurements of skin temperature can also serve direct or indirect indications of core body temperature as a core vital sign. Integration with, or operation as heat sources, allows temperature sensors to be used to assess local thermal transport characteristics, as indicators of hydration status or microvascular perfusion, with applications in diagnosis and monitoring of cutaneous diseases and wound healing. Arrays of heaters and sensors can also be used to quantitatively measure flow rates of blood through large, near-surface blood vessels, and cerebrospinal fluid through ventriculoperitoneal shunts. In all cases, physics-based models define optimal layouts and modes of operation. Multimodal methods that exploit multiple different measurement techniques allow a single device to output various different parameters simultaneously (e.g., *T*, *k*, *v*, and *w*). Wireless data transfer capabilities permit free, untethered use in non-clinical environments, as alternatives to traditional optical and imaging techniques used for the analysis of vascular and tissue health. The various thermal sensing technologies outlined here allow for precision, low-cost measurements. Development of soft, epidermal devices greatly enhance applicability and use of such devices for both clinical and non-clinical environments.

Distilling the individual contributions of perfusion and macrovascular flow to the measured thermal responses, especially within physiological regions where both venous and capillary flow are significant, remains a challenge. Convective flow from *v* or *w* both influence Δ*T* or Δ*q*. Spatiotemporal mapping offers the greatest potential in capturing both the magnitude and direction of flow. Techniques like those found in Ref. 151 offer the ability to compute *v* and *w* using wearable device form factors, while optical mechanisms, such as LCSI offer alternate, non-wearable approaches to accomplish similar goals.

Combining thermal sensors with other complementary methods, such as sweat analytics, skin pH, transepidermal water loss, strain, and modulus is another important future direction for applications in cutaneous and systemic disease diagnosis and monitoring. For example, Lee *et al.* (2016) developed a graphene-based electrochemical device with thermo-responsive microneedles for monitoring and therapy of diabetes.^233^ The device contains a heater, and temperature, humidity, tremor (strain), glucose, and pH sensors, along with polymer microneedles that can deliver drugs transcutaneous upon thermal activation. This suite of sensors is essential for monitoring the pathogenesis of diabetes and for enabling closed-loop feedback control.

Programmable thermal therapy, which refers to the controlled delivery of heat to the surface of the skin at a desired temperature and duration, is another area of interest, where heat induces local vasodilation and thermal expansion of collagen tissue that can reduce swelling, muscle pain, and alleviate numbness.^234^ Wearable embodiments entail Joule heating of metal nanoparticles or other conductive nanomaterials embedded in elastomers. Stretchable, thin films of metal patterned into filamentary mesh/fractal geometries represent an additional option for wearable heaters.^235^ A recent work in flexible, stretchable, wearable thermotherapy bands and patches illustrates the ability to provide controlled heat to the skin for such purposes.^234,236^ Such heating can also be used to study hemodynamic properties and tissue condition, including responsiveness to applied thermal stimuli as an indicator of vascular health.^237^ Thermally induced changes in biological properties of the skin and individual cell types also merit further study.

While this review focuses on noninvasive thermal measurements of the properties of the skin from its surface, injectable and implantable thermal sensors are also important, not only for skin but for underlying organs as well. A recent work (2022) demonstrates, for example, the use of wireless, implantable thermal probes for measuring changes in perfusion of skin flaps.^215^ Injectable thermal sensors and “smart” balloon catheters can assist in cardiac ablation procedures.^238,239^ This emerging field of implantable sensors for measurements of the thermophysical properties of internal organs holds great promise not only in monitoring disease progression but also in understanding disease mechanisms. Ultimately, measurements of the thermal properties of the skin and other tissues have great potential to impact our understanding of biology and medicine. This area of research combines fundamental aspects of measurement physics with practical opportunities in translation medicine, in a manner that suggests a vibrant future.

## Data Availability

Data sharing is not applicable to this article as no new data were created or analyzed in this study.
